# A maximum-type microbial differential abundance test with application to high-dimensional microbiome data analyses

**DOI:** 10.3389/fcimb.2022.988717

**Published:** 2022-10-28

**Authors:** Zhengbang Li, Xiaochen Yu, Hongping Guo, TingFang Lee, Jiyuan Hu

**Affiliations:** ^1^ School of Mathematics and Statistics, Central China Normal University, Wuhan, China; ^2^ School of Mathematics and Statistics, Hubei Normal University, Huangshi, China; ^3^ Division of Biostatistics, Department of Population Health, New York University (NYU) Grossman School of Medicine, New York, NY, United States

**Keywords:** microbiome data, relative abundances, high-dimensional compositional, differential abundance analysis, sparse alternatives

## Abstract

**Background:**

High-throughput metagenomic sequencing technologies have shown prominent advantages over traditional pathogen detection methods, bringing great potential in clinical pathogen diagnosis and treatment of infectious diseases. Nevertheless, how to accurately detect the difference in microbiome profiles between treatment or disease conditions remains computationally challenging.

**Results:**

In this study, we propose a novel test for identifying the difference between two high-dimensional microbiome abundance data matrices based on the centered log-ratio transformation of the microbiome compositions. The test p-value can be calculated directly with a closed-form solution from the derived asymptotic null distribution. We also investigate the asymptotic statistical power against sparse alternatives that are typically encountered in microbiome studies. The proposed test is maximum-type equal-covariance-assumption-free (MECAF), making it widely applicable to studies that compare microbiome compositions between conditions. Our simulation studies demonstrated that the proposed MECAF test achieves more desirable power than competing methods while having the type I error rate well controlled under various scenarios. The usefulness of the proposed test is further illustrated with two real microbiome data analyses. The source code of the proposed method is freely available at https://github.com/Jiyuan-NYU-Langone/MECAF.

**Conclusions:**

MECAF is a flexible differential abundance test and achieves statistical efficiency in analyzing high-throughput microbiome data. The proposed new method will allow us to efficiently discover shifts in microbiome abundances between disease and treatment conditions, broadening our understanding of the disease and ultimately improving clinical diagnosis and treatment.

## 1 Introduction

The human microbiota, a collection of microbes living on or inside human bodies, has been shown to play a fundamental role in human health and diseases, including diabetes, cancer, and obesity (Turnbaugh et al. (2007); Ursell et al. (2012)). Recently, the metagenomic next-generation sequencing (mNGS) technique has been introduced in the clinical diagnosis of infectious diseases (Gu et al. (2019); Dulanto Chiang and Dekker (2020); Govender et al. (2021)) and emerged as a revolutionary technique to replace/supplement traditional culture-based and molecular microbiologic techniques: i) mNGS allows the parallel sequencing of hundreds of samples per run; ii) it provides an unbiased detection of bacteria, viruses, fungi, and parasites collectively; iii) this culture-free technology enables the identification of new species and others.

In microbiome studies, it is of general research interest to study the microbiome profiles/features between different disease treatments or conditions. Various statistical methods have been proposed recently for examining differential abundances (DAs) (Anderson (2014); Cao et al. (2018); Zhao et al. (2018); Banerjee et al. (2019); Lin and Peddada (2020)). These methods can be categorized into univariate and multivariate approaches depending on whether microbial features are analyzed individually or in a set-based fashion. For example, Lin and Peddada (2020) proposed ANCOM-BC under a linear regression framework to conduct DA analysis for the assessed taxa individually. DESeq2 (Love et al. (2014)) and edgeR (Robinson et al. (2010)), two popular differential expression gene analysis methods, are commonly used for differential abundance analysis. However, multiple comparison procedures need to be conducted afterward for these univariate methods, which largely hinders the statistical power (Hu et al. (2018)). Alternatively, we can assess the microbial features as a set in order to enhance the statistical power. Typically, the microbial abundances are normalized toward the total counts to make the microbial proportions [or called relative abundances (RAs)] comparable between samples. The normalized data have a summation of the features equal to one, termed compositional in microbiome studies (Mandal et al. (2015); Gloor et al. (2017)). Directly applying standard multivariate statistical methods developed for unconstrained data to compositional data may result in inappropriate or misleading inferences. Cao et al. (2018) proposed a two-sample test for assessing the difference between two high-dimensional microbial composition matrices and treating all microbial features (the microbiome profile) as a set. Banerjee et al. (2019) proposed an adaptive test for comparing microbiome compositions from two independent groups. Zhao et al. (2018) developed a generalized Hotelling test for paired microbiome composition data comparison. These methods can be applied to the full microbiome profiles and also microbial features that belong to the same upper-level taxonomic rank, gene family, or functional pathway. Nevertheless, they either need a strong assumption that the covariance matrices of compared compositions are equal (Cao et al. (2018)) or require time-consuming permutations to determine the statistical significance (Zhao et al. (2018); Banerjee et al. (2019)).

To address this challenge, we propose a two-sample maximum-type equal-covariance-assumption-free (MECAF) test. This multivariate differential abundance test statistics relaxes the equal covariance assumption required by the test proposed by Cao et al. (2018). The closed-form formula of the asymptotic null distribution largely resolves the computational burden in microbiome analysis. The method can be applied to analyze both taxonomic and functional profiles including microbial taxa (operational taxonomic units (OTUs), strains, etc., from either shotgun metagenomic or 16S rRNA amplicon sequencing technique), functional pathways, and gene families. The performance of the proposed MECAF test is demonstrated through simulation studies and applications to the shotgun metagenome sequencing study of *Clostridium difficile* infection (CDI) (Vincent et al. (2016)) and the 16S rRNA amplicon murine microbiome study of type I diabetes (T1D) (Livanos et al. (2016)).

The rest of this article is as follows. In Section 2, we briefly introduce the novel test statistics MECAF for conducting a two-group comparison of microbiome compositions, carry out extensive simulations to estimate the empirical type I error rate and statistical power for the proposed test in comparison with competing methods, and further conduct two real data applications. We conclude with a discussion in Section 3. Notation, test hypothesis, and the asymptotic properties of the MECAF test are given in the last section. All the theoretical derivations are detailed in the **Supplementary Material**.

## 2 Results

### 2.1 The MECAF test

We consider the comparison of high-dimensional microbiome compositions from two independent groups. We propose an independent two-sample test named MECAF, which 1) is derived based on the centered log-ratio (CLR) transformed compositions, 2) has the aim to test the null hypothesis of equal mean vectors for the microbial features against unequal mean vectors, and 3) does not require the assumption of equal covariance matrices between groups. The equation of the test statistics and corresponding asymptotic null distribution is given in Section 4.

### 2.2 Simulation studies

#### 2.2.1 Simulation setup

We conducted extensive simulations to evaluate the numerical performance of the proposed MECAF test compared with competing methods under various scenarios. The simulation parameters were set up similarly to those in Cao et al. (2018) for the case of two independent samples in order to generate microbiome composition data. The log transformation of microbiome absolute abundance data *L* ^1^ and *L* ^2^ was first generated from the multivariate Gaussian distribution by assuming that $L_{i}^{1}\overset{i.i.d.}{∼}N\left(\mu_{L}^{1},{\text{Σ}}_{L}^{1}\right)$ and $L_{i}^{2}\overset{i.i.d.}{∼}N\left(\mu_{L}^{2},{\text{Σ}}_{L}^{2}\right)$ . Then the raw absolute abundance *A* ^1^, *A* ^2^, relative abundances *R* ^1^, *R* ^2^, and CLR transformation of RA matrices *X* ^1^, *X* ^2^ can be generated accordingly with certain transformations detailed in the Methods section. We specify the location and covariance parameters for distributions $N\left(\mu_{L}^{1},{\text{Σ}}_{L}^{1}\right)$ and $N\left(\mu_{L}^{2},{\text{Σ}}_{L}^{2}\right)$ detailed as follows so that simulation data matrices can be generated with various covariance structures under the null and alternative hypotheses.

- Specification of location parameters $\mu_{L}^{1}$ and $\mu_{L}^{2}$ . Following Cao et al. (2018), the components of $\mu_{L}^{1}$ were drawn from the uniform distribution Uniform (0,10). Each component of $\mu_{L}^{2}$ was set by $\mu_{L:j}^{2}=\mu_{L:j}^{1}-\delta_{j}\sigma_{L:jj}\frac{1}{2}{\left(\frac{log\;p}{n}\right)}^{\frac{1}{2}}$ . Here, *δ_j_* represents the signal, i.e., the difference in CLR means for component *j* between two groups. *s*=⌞0*p*⌟,⌞0.05*p*⌟,⌞0.1*p*⌟⌞0.2*p*⌟ , and ⌞0.2*p*⌟ components (taxa) and randomly chosen from *p* components to be the signal taxa and the corresponding *σ_j_* ‘s were randomly drawn from $\text{Uniform}\left[-2\sqrt{2},2\sqrt{2}\right]$ . The other *σ_j_* ‘s were set as 0. We can see that *s*= ⌞0*p*⌟ corresponds to the null hypothesis setting and *s*= ⌞0.05*p*⌟,⌞0.1*p*⌟,⌞0.2*p*⌟ represent three alternative hypothesis settings. When *s* becomes larger, there are more signal taxa in the microbiome compositions. *σ_L_*:*_jj_* is the *j*th diagonal component of the covariance matrix ${\text{Σ}}_{L}^{2}$ with specifications as follows.
- Specification of covariance matrices ${\text{Σ}}_{L}^{1}$ and ${\text{Σ}}_{L}^{2}$ . We included two types of covariance matrices, i.e., a banded covariance matrix Σ*_B_* and sparse covariance matrix Σ*_S_* with the same parameters as those in Cao et al. (2018). Three scenarios were considered to assess the impact of equal *vs.* unequal covariance matrices between two groups in the comparison of compositional mean vectors. Specifically, in Scenario 1, the covariance matrices of groups 1 and 2 are set as ${\text{Σ}}_{L}^{1}={\text{Σ}}_{B}$ , and ${\text{Σ}}_{L}^{2}={\text{Σ}}_{S}$ to represent the setting with unequal covariance matrices between groups; equal banded covariance matrices were considered in Scenario 2, i.e., ${\text{Σ}}_{L}^{1}={\text{Σ}}_{L}^{2}={\text{Σ}}_{B}$ ; and equal sparse covariance matrices were considered in Scenario 3, i.e., ${\text{Σ}}_{L}^{1}={\text{Σ}}_{L}^{2}={\text{Σ}}_{S}$ .

#### 2.2.2 Competing methods

In this article, we mainly focus on the comparisons of multivariate differential abundance approaches. By assuming that covariance matrices for the CLR of compositions are equal, i.e., ${\text{Σ}}_{X}^{1}={\text{Σ}}_{X}^{2}$ , Cao et al. (2018) proposed a test for hypothesis (1) as $T_{\text{MEC}}=\underset{1\leq j\leq p}{\max\;}{\left(\sqrt{\frac{n_{1}n_{2}}{n_{1}+n_{2}}}\frac{{\bar{X}}_{j}^{1}-{\bar{X}}_{j}^{2}}{\sqrt{{\hat{\sigma }}_{0:jj}}}\right)}^{2}$ , where ${\hat{\sigma }}_{0:jj}=\frac{1}{n_{1}+n_{2}}\left[\overset{i=1}{\underset{n_{1}}{\sum^{}}}{\left(X_{ij}^{1}-{\bar{X}}_{j}^{1}\right)}^{2}+\overset{i=1}{\underset{n_{2}}{\sum^{}}}{\left(X_{ij}^{2}-{\bar{X}}_{j}^{2}\right)}^{2}\right]$ . Since this is a maximum-type test with an equal covariance assumption, we denote it as the MEC test in this article. In addition, we also assessed the performance of the MEC statistics applied to the raw RA, the log transformation of RA, and the original AA. These three tests are obtained by replacing the CLR data used by MEC, i.e., *X^g^*, with *R^g^*, log (*R^g^*), and *A^g^*,*g* = 1,2, denoting by MEC-Raw, MEC-Log, and MEC-Oracle, respectively. MEC-Oracle is considered the benchmark method in the simulation study (under equal covariance matrices assumption), as the true difference is simulated for the log-absolute abundances. Permutational multivariate analysis of variance (PERMANOVA) is a popular multivariate analysis method widely adopted in community-level microbiome data analysis (Anderson (2014)). We therefore included PERMANOVA, which tests the null hypothesis that the centroid and the spread of the microbiome profiles are equivalent for the compared groups.

We set the sample size in the first group as *n*_1_ = 100 and increased the sample size in the second group *n*_2_ from 200 to 300. We increased the number of components (taxa) *p* 100 to 300 to demonstrate different relationships between *n* = *n*_1_ + *n*_2_ and *p*. We set the significance level as *α* = 0.05 in the simulation, and 1,000 replications were conducted to evaluate the empirical type I error rate and statistical power of the assessed methods under various settings.

#### 2.2.3 Simulation results

**Figure 1:**
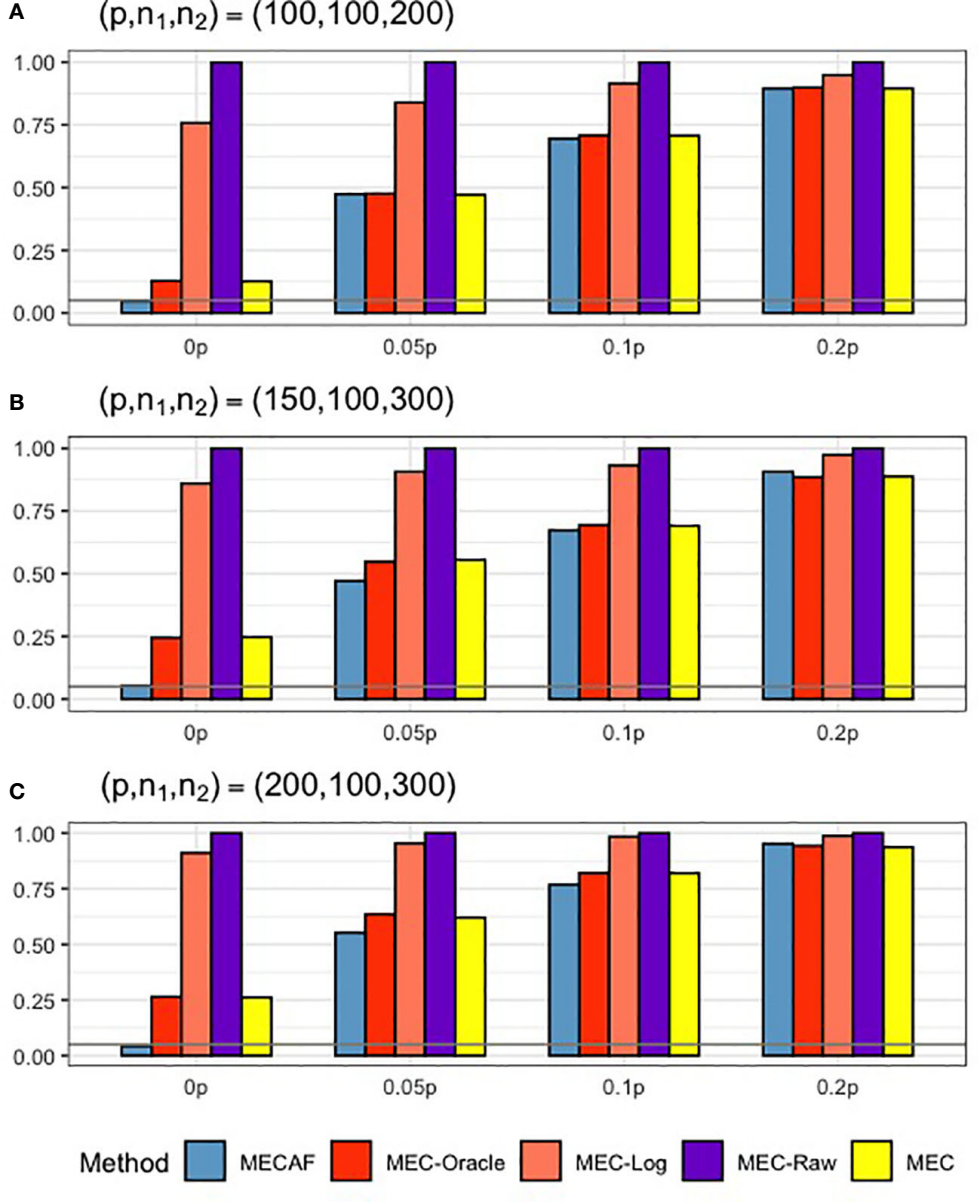
Simulation results for Scenario 1: unequal covariance matrices between compared groups. The empirical type I error rate (H_0: 0p) and statistical power under three sparsity measures (*H_a_*:0.05*p*, 0.1*p*, and 0.2*p*) for MECAF and competing methods MEC-Oracle, MEC-Log, MEC-Raw, and MEC. A horizontal line with *α* = 0.05 indicates the significance level. The number of taxa and sample sizes were set as follows: **(A)** (*p*,*n*_1_,*n*_2_) = (100,100,200); **(B)** (*p*,*n*_1_,*n*_2_) = (150,100,300); **(C)** (*p*,*n*_1_,*n*_2_) = (200,100,300).

**Figure 1** shows the numerical performance of assessed methods under Scenario 1, where unequal covariance matrices are considered. All competing methods that require equal covariance matrix assumption, i.e., MEC-Oracle, MEC-Log, MEC-Raw, and MEC, have inflated type I error rates. The type I error rate of MEC approaches 0.25 when *p* = 150 and *p* = 200. This indicates that MEC-type tests are not applicable to data with unequal matrices. In comparison, the proposed MECAF test can control the empirical type I error rate around the nominal level of 0.05. The statistical power of MECAF increases with the proportion of signal taxa. The results of PERMANOVA are not shown in **Figure 1**, since the corresponding type I error rate and statistical power are all equal to 1 under this scenario. This is because the abundance data were generated from unequal covariance matrices and therefore violate the null hypothesis tested by PERMANOVA.

**Figure 2:**
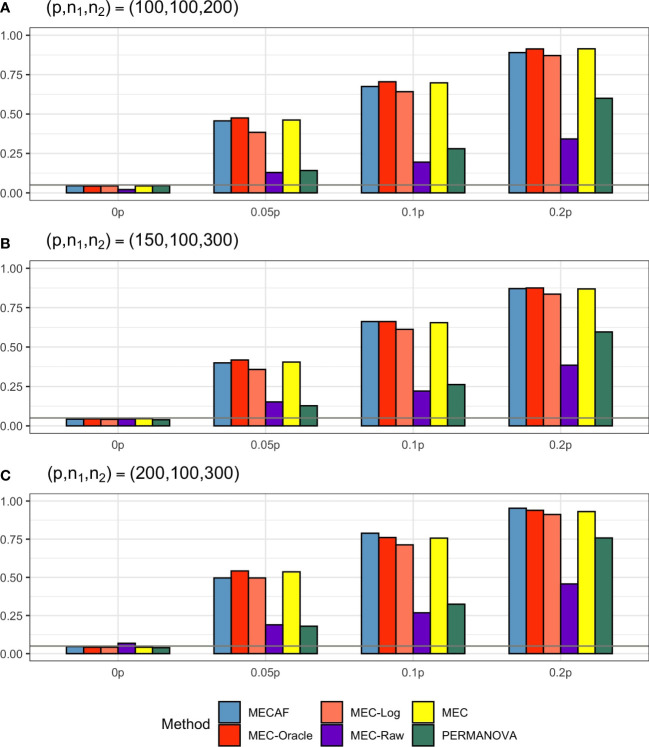
Simulation results for Scenario 2: same banded covariance matrix between compared groups. The empirical type I error rate (H_0: 0p) and statistical power under three sparsity measures (*H_a_*:0.05*p*, 0.1*p*, and 0.2*p*) for MECAF and competing methods MEC-Oracle, MEC-Log, MEC-Raw, and MEC. A horizontal line with *α* = 0.05 indicates the significance level. The number of taxa and sample sizes were set as follows: **(A)** (*p*,*n*_1_,*n*_2_) = (100,100,200); **(B)** (*p*,*n*_1_,*n*_2_) = (150,100,300); **(C)** (*p*,*n*_1_,*n*_2_) = (200,100,300).

**Figure 3:**
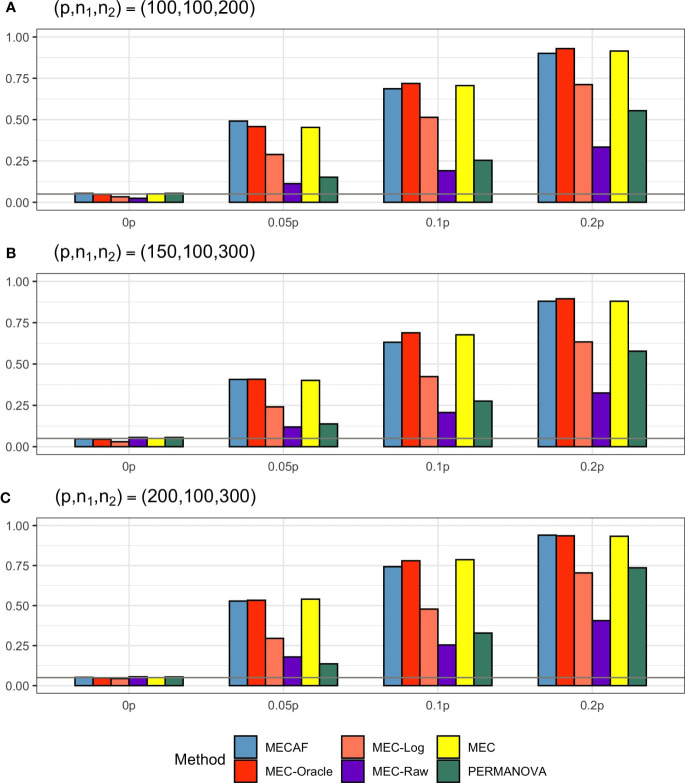
Simulation results for Scenario 3: same sparse covariance matrix between compared groups. The empirical type I error rate (H_0: 0p) and statistical power under three sparsity measures (*H_a_*:0.05*p*, 0.1*p*, and 0.2*p*) for MECAF and competing methods MEC-Oracle, MEC-Log, MEC-Raw, and MEC. A horizontal line with *α* = 0.05 indicates the significance level. The number of taxa and sample sizes were set as follows: **(A)** (*p*,*n*_1_,*n*_2_) = (100,100,200); **(B)** (*p*,*n*_1_,*n*_2_) = (150,100,300); **(C)** (*p*,*n*_1_,*n*_2_) = (200,100,300).

Simulation results for equal banded covariance and equal sparse covariance scenarios are depicted in **Figures 2** and **3**, respectively. As expected, all assessed methods have a well-controlled type I error rate under these simulation settings. MECAF and MEC achieved statistical power comparable to that of MEC-Oracle, with sparsity measure *s* ranging from ⌞0.05*p*⌟⌞0.2*p*⌟ to ⌞0.2*p*⌟. This indicates the statistical efficiency of the MECAF test. In comparison, MEC-Log, MEC-Raw, and PERMANOVA have evidently smaller power than MEC-Oracle for all settings of the two scenarios.

In summary, MECAF has a well-controlled type I error rate for two group comparisons of mean composition vectors either with equal or unequal covariance matrices. The statistical power is desirable under all scenarios with various sparsity measures.

### 2.3 Applications to two microbiome studies

Here, we first apply the proposed MECAF test to the shotgun metagenomic sequencing data from the CDI study (Vincent et al. (2016)). Since the test is also applicable to microbiome abundance data generated from the 16S rRNA amplicon sequencing technology, we further illustrate our proposed method in a murine microbiome study of T1D [Livanos et al. (2016)].

#### 2.3.1 Analysis of the *Clostridium difficile* infection metagenomic dataset

Vincent et al. (2016) conducted a prospective study to investigate the intestinal microbiota dynamics over time among 98 hospitalized patients at risk for CDI, a leading infectious cause of nosocomial diarrhea. Patients were followed up to 60 days, and a total of *N* = 229 fecal samples (averaging 2.34 samples per subject) were examined by the shotgun metagenomics sequencing platform. The bioinformatics pre-processing steps were detailed by Vincent et al., 2016, and the processed microbial counts and metadata are available in the R package “curatedMetagenomicsData” (Version 1.16.1) from the Bioconductor by running the function *curatedMetagenomicData(‘VincentC_2016.metaphlan_bugs_list.stool’,dryrun = FALSE)* (Pasolli et al. (2017)). Zero counts were imputed with 0.5 before converting counts to relative abundances for taxa from taxonomic ranks of phylum, class, order, family, genus, and species (strains). In this secondary data analysis, we aim to examine whether there are shifts in the microbial relative abundances between patients with CDI or asymptomatic *C. difficile* colonization (CDI group, N = 8 subjects) and patients without (control group, N = 90 subjects) i) upon hospitalization (baseline), ii) at 1 week of hospitalization, and iii) over 1 week of hospitalization. The latest sample at each time window was included for each patient.

**Figure 4:**
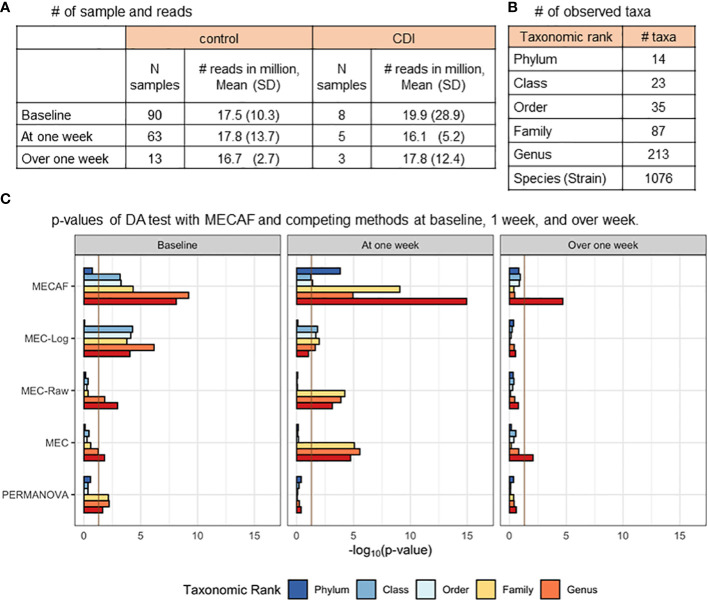
DA analysis of the CDI metagenomic dataset. **(A)** The number of samples and microbial reads are summarized. **(B)** Number of observed taxa at each of the taxonomic ranks. **(C)** p-Values of DA test with MECAF and competing methods at baseline, 1 week, and over 1 week. p-Values were –log_10_ transformed to better illustrate the statistical significance where the vertical line of -log_10_0.05 indicates a p-value equal to 0.05. DA, differential abundance; CDI, *Clostridium difficile* infection.

**Figure 4** shows the available samples at each assessed time window (**Figure 4A**), the number of taxa observed at each taxonomic rank (**Figure 4B**), the differential abundance test results of the MECAF test, and competing methods (**Figures 4C**). A p-value of<0.05 was indicated as statistically significant. Of note, MEC-Oracle was not included in the comparison since the absolute abundance data required by MEC-Oracle are unobservable in real data. The results of MECAF indicate significant differences in the microbiome compositions between CDI and control patients at baseline and week 1. The significance is consistent for most of the taxonomic ranks, and a stronger signal is depicted at the lower ranks. The difference though seems to disappear after 1 week of hospitalization, where only the test at the species (strain) level is significant. In comparison, MEC does not detect significant differences in microbiome compositions except at the species and strain levels at baseline and over 1 week, with less stringent p-values reported. At week 1, MEC detects microbial composition differences at the family, genus, and species (strain) levels but not at the phylum, class, or order level. MEC-Raw and PERMANOVA have similar results as MEC, and MEC-log reported similar results as those of MECAF with higher p-values. In summary, we observe more consistent findings from the MECAF test over six taxonomic ranks. The corresponding p-values are in general smaller than those of competing methods, indicating statistical efficiency gain over other methods.

We further assessed the type I error rate of competing methods using the baseline data of the control subjects (*N* = 90). To achieve this, we randomly split the dataset into two groups and conducted DA tests between the mock groups. A total of 1,000 replications were carried out to calculate the empirical type I error rate as shown in **Table 1**. As expected, all assessed methods are able to control the type I error rate below the nominal level of 0.05.

**Table 1:** Empirical type I error rate with real data from the CDI study.

Method	Taxonomic rank					
	Phylum	Class	Order	Family	Genus	Strains
MECAF	0.033	0.045	0.038	0.032	0.023	0.016
MEC	0.032	0.044	0.038	0.032	0.023	0.016
MEC-Log	0.029	0.041	0.031	0.035	0.021	0.013
MEC-Raw	0.011	0.013	0.002	0.003	0.003	0.000
PERMANOVA	0.049	0.056	0.046	0.051	0.052	0.048

CDI, *Clostridium difficile* infection.

#### 2.3.2 Analysis of the MICE 16S rRNA amplicon microbiome data

Livanos et al. (2016) carried out a murine microbiome study to investigate the effect of early-life antibiotic exposure on the alteration of gut microbiota composition. Here, we re-examined the 16S microbiome abundance profile from the early-life sub-therapeutic antibiotic treatment (STAT) group and the control group that received no antibiotic exposure. The abundances were compared between the two groups at each of the four assessed time points, i.e., weeks 3, 6, 10, and 13, for female and male mice using the MECAF test and competing methods.

**Figure 5:**
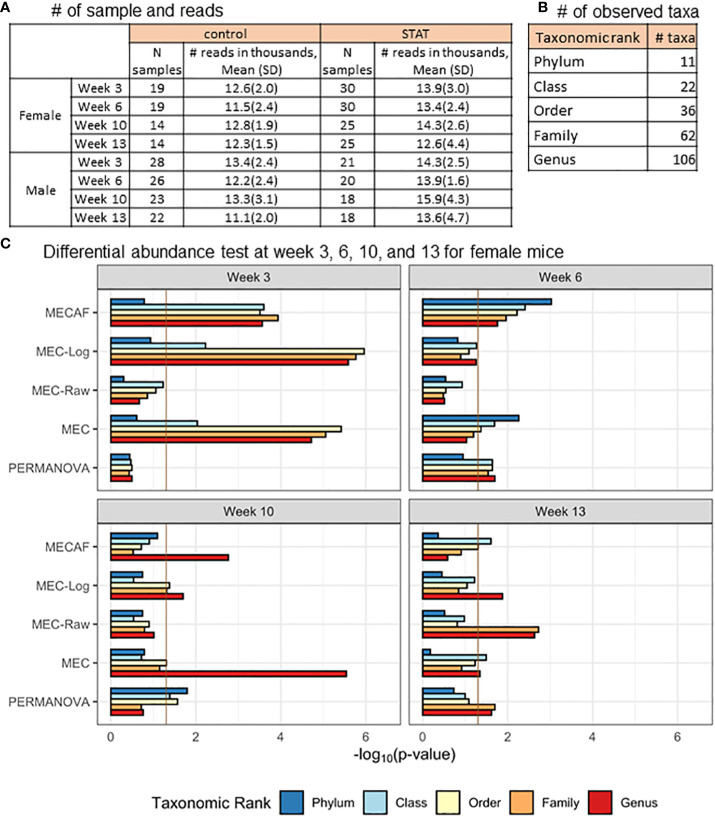
DA analysis in female mice of the murine microbiome study. **(A)** The number of samples and microbial reads are summarized separately for female and male mice. **(B)** Number of observed taxa at each of the taxonomic ranks. **(C)** p-Values of DA test with MECAF and competing methods at four assessed time points. p-Values were –log_10_ transformed to better illustrate the statistical significance where the vertical line of -log_10_0.05 indicates the significance level. DA, differential abundance.

**Figure 6:**
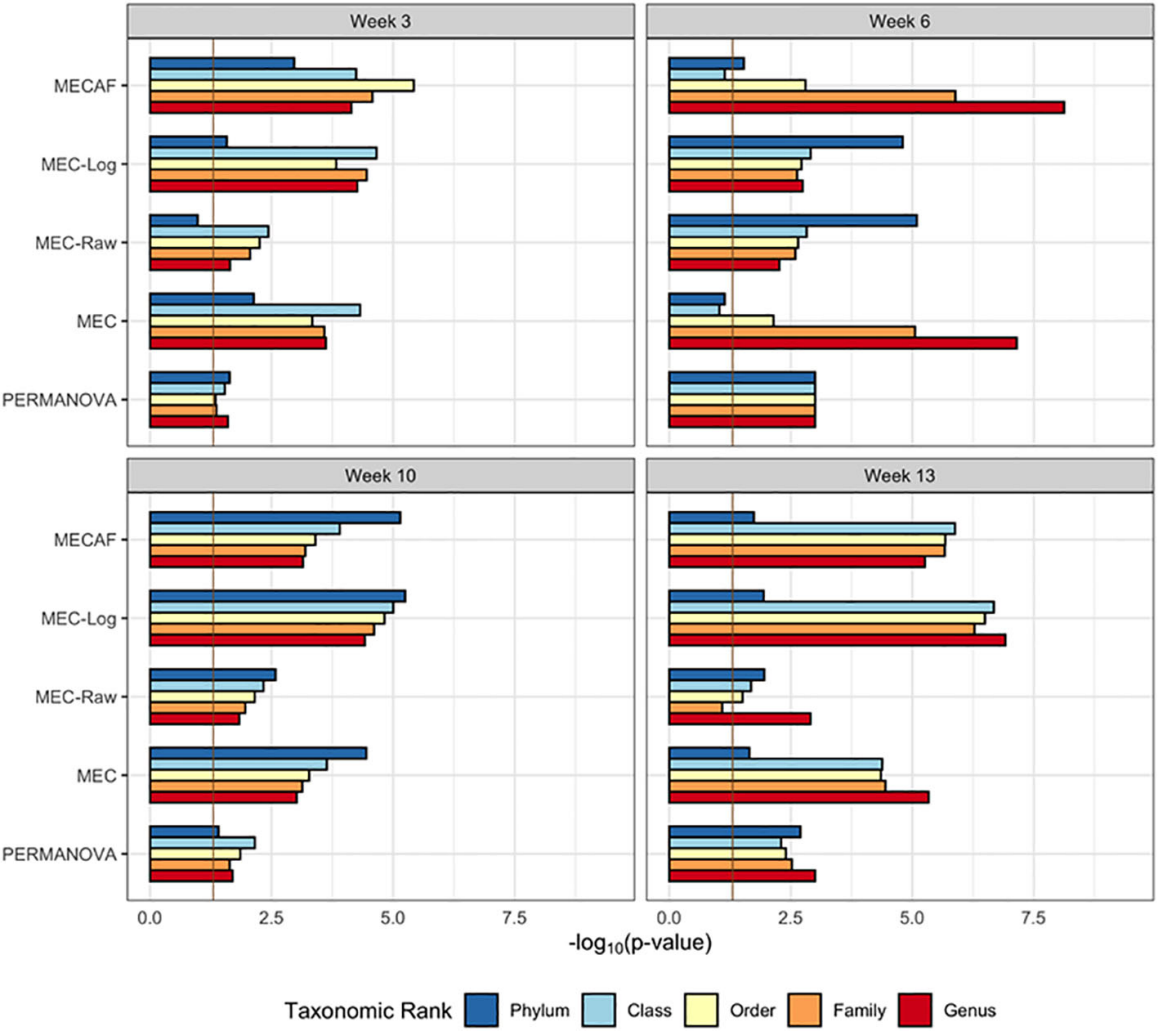
DA analysis in male mice of the murine microbiome study. p-Values of DA test with MECAF and competing methods at four assessed time points are shown. p-Values were –log_10_ transformed to better illustrate the statistical significance where the vertical line of -log_10_0.05 indicates the significance level. DA, differential abundance.

The available samples and number of taxa observed are shown in **Figures 5A, B**, which illustrate the circumstance of *n*< *p* (number of samples< number of taxa) most often encountered in microbiome data analysis. The differential abundance analysis results from the phylum to genus rank are depicted in **Figures 5C**, **6** for female and male mice, respectively. The result from MECAF indicated that in female mice, the abundance profile is significantly different in the STAT group from the control group from week 3 to 6 for almost all taxonomic ranks. The significance is weaker at weeks 10 and 13, indicating the recovery of gut microbiota in the STAT mice upon maturation. In comparison, a significant difference is detected by MECAF in male mice over the four assessed time points. This result is consistent with Livanos et al. (2016) in which the alpha- and beta-diversity measures were compared between groups over time. MEC has similar results to MECAF. MEC-Log did not detect significance in female mice from weeks 6 to 10 for most of the taxonomic ranks. MEC-Raw and PERMANOVA either did not detect significant differences (female mice from weeks 3 to 10) or reported weaker signals (male mice, weeks 3, 10, and 13).

## 3 Discussion

In this article, we propose a novel test named MECAF for the two-sample test of high-dimensional compositions. The test statistics is developed based on the centered log-ratio transformation of the compositions following Aitchison (1982) and Cao et al. (2018). The asymptotic null distribution of the test statistic is derived, and the power against sparse alternatives is investigated. The derived null distribution allows for the closed-form solution of statistical significance and largely resolves the computational burden. Simulation results show that the proposed method is evidently more powerful than competing methods when the covariance matrices differ between groups, and comparable performance is achieved when the groups have equal covariance. Two real data applications have illustrated the usefulness of the proposed method.

The comparisons of competing methods have focused on multivariate approaches only since they are not directly comparable with univariate approaches (such as ANCOM-BC, DESeq2, and edgeR). We admit the limitation of the MECAF test that it is used as the first screening step of microbiome analysis for the examination of the global shift of microbiome profiles. Other regression models that are built upon Dirichlet distribution or generalized Dirichlet distribution [Tang and Chen (2019); Liu et al. (2020)] have distinct features from differential abundance methods discussed herein. For example, they allow for covariate adjustment, feature selections, repeated sampling, etc., which is beyond the scope of this article.

The MECAF test extends the MEC proposed by Cao et al. (2018) by relaxing the assumption of equal covariance matrix structure between groups. Therefore, MECAF can be applied to a wider set of circumstances. In the real data applications, we applied MECAF to compare the microbiome abundances aggregated to each taxonomic rank. In practice, we can also apply MECAF to assess the composition of a given sub-tree or a subset of the microbiome taxa (Shi and Li (2017)). As a future direction, we will aim to extend the MECAF test to accommodate repeated measures from each individual for group comparisons.

## 4 Methods

### 4.1 Notation and specification of test hypothesis

In this article, we consider microbiome compositions from two independent groups. The notation used in this manuscript is summarized in **Table 2**. Specifically, for subject *i* from group *g*(*g* = 1,2), denote the *n_g_* independently observed composition vectors as $\left\{R_{i}^{g}={\left(R_{i1}^{g},\ldots ,R_{ip}^{g}\right)}^{⊤},i=1,2,\ldots ,n_{g}\right\}$ with length of *p*, and the *j*th component (taxon) of the vector $R_{i}^{g}$ as $R_{ij}^{g}$ , where $R_{ij}^{g}\in \left(0,1\right)$ . Of note, zero proportions are imputed by a pseudo-positive proportion prior to conducting the analysis. Then the compositional constraints can be expressed as $\sum_{j=1}^{p}R_{ij}^{g}=1,i=1,\ldots ,n_{g};g=1,2$ . Obviously, $R_{i}^{g}$ represents compositions that lie in the *p* - 1 dimensional simplex $\left\{S^{p-1}=\left(r_{1},\ldots ,r_{p}\right):r_{j}>0,j=1,\ldots ,p,\sum_{j=1}^{p}r_{j}=1\right\}$ . *R* ^1^ and *R* ^2^ are the observed data matrices of dimension *n*_1_ × *p* and *n*_2_ × *p*, respectively, from the two groups. Let $\left\{A_{i}^{g}={\left(A_{i1}^{g},\ldots ,A_{ip}^{g}\right)}^{⊤},i=1,\ldots ,n_{g}\;g=1,2\right\}$ denote the *n_g_* unobserved absolute abundances of the microbiome. The numerical relationship between the absolute abundance matrix and composition matrix is as follows:

**Table 2:** Notation summary.

Notation	Description
*g*	Group indicator, *g* = 1,2
*n_g_*	Sample size for group *g*
*i*	*i*th sample, *i* = 1,2,…,*n*_1_ for group 1, and *i* = 1,2,…,*n*_2_ for group 2
*p*	Number of taxa in the microbiome data matrix
*R_g_*	Observed microbial relative abundances (RAs) for group *g* with dimension *n_g_* × *p*
$R_{i}^{g}$	Observed RA for subject *i* for group *g*
*A^g^*	Unobserved microbial absolute abundances (AAs) for group *g* with dimension *n_g_* × *p*
*L^g^*	Unobserved log transformation of AA (log-AA) for group *g* with dimension *n_g_* × *p*
$L_{i}^{g}$ , $\mu_{L}^{g}$ , and $\Sigma_{L}^{g}$	Unobserved log-AA for subject *i* from group *g*, and $L_{i}^{g}i.i.d.$ from distribution with mean $\mu_{L}^{g}$ and covariance matrix $\Sigma_{L}^{g}$
*X^g^*	Observed centered log-ratio transformation of relative abundances of group *g* with dimension *n_g_* × *p*
$X_{i}^{g}$ , $\mu_{X}^{g}$ , and $\Sigma_{X}^{g}$	Observed centered log-ratio (CLR) transformation of RA for subject *i* from group *g*, and $X_{i}^{g}i.i.d.$ from distribution with mean $\mu_{X}^{g}$ and covariance matrix $\Sigma_{X}^{g}$


$$R_{ij}^{g}=\frac{A_{ij}^{g}}{\sum_{j=1}^{p}A_{ij}^{g}},i=1,\ldots ,n_{g};j=1,\ldots ,p;g=1,2,$$

where $A_{ij}^{g}$ is the *j*th component of $A_{i}^{g}$ . Suppose the log transformations of $A_{i}^{g}$ , denoted by $L_{i}^{g}$ , are i.i.d. from distributions with mean vectors $\mu_{L}^{g}={\left(\mu_{L:1}^{g},\ldots ,\mu_{L:p}^{g}\right)}^{⊤}=E[L^{g}]$ and covariance matrices ${\text{Σ}}_{L}^{g}={\left(\sigma_{L:kj}^{g}\right)}_{k,j=1,\ldots ,p}=\text{cov(}L^{g},L^{g}),g=1,2$ . Cao et al. (2018) introduced a testable hypothesis to compare the log-mean absolute abundance vectors through the observed compositional data *R* ^1^ and *R* ^2^ by exploiting the CLR transformation of the compositions. Denote the CLR transformation of $R_{ij}^{g}$ by


$$X_{ij}^{g}=\log\;\left(\frac{R_{ij}^{g}}{{\left(\prod_{j=1}^{p}R_{ij}^{g}\right)}^{1/p}}\right),i=1,\ldots ,n_{g};j=1,\ldots ,p;g=1,2.$$

Assume that the CLR vectors $\left\{X_{i}^{g}={\left(X_{i1}^{g},\ldots ,X_{ip}^{g}\right)}^{⊤},i=1,2,\ldots ,n_{g}\right\}$ are i.i.d. from distributions with mean vectors $\mu_{X}^{g}={\left(\mu_{X:1}^{g},\ldots ,\mu_{X:p}^{g}\right)}^{⊤}=E[X^{g}]$ , and covariance matrices ${\text{Σ}}_{X}^{g}={\left(\sigma_{X:kj}^{g}\right)}_{k,j=1,\ldots ,p}=\text{cov(}X^{g},X^{g}),g=1,2.$ Then the testable hypothesis under the definition of compositional equivalence [please see Definition 1 in Cao et al. (2018)] is


(1) $$H_{0}^{\left(1\right)}:\mu_{X}^{1}=\mu_{X}^{2}\text{ versus }H_{1}^{\left(1\right)}:\mu_{X}^{1}\neq \mu_{X}^{2}.$$

In this work, we consider another testable hypothesis of compositional equivalence as follows. It is straightforward that $\sum_{j=1}^{p}\mu_{X:j}^{1}=0$ , and $\sum_{j=1}^{p}\mu_{X:j}^{2}=0$ , $\mu_{X}^{1}=\mu_{X}^{2}$ holds if and only if for *j*∈{1,…,*p*−1} , as $\mu_{X:j}^{1}=\mu_{X:j}^{2}$ . Therefore, an equivalent hypothesis that only considers the first p - 1 components is


(2) $$H_{0}^{\left(2\right)}:\mu_{X:j}^{1}=\mu_{X:j}^{2}\;for\;any\;j\in \left\{1,\ldots ,p-1\right\}\text{ versus }H_{1}^{\left(2\right)}:\mu_{X:j}^{1}\neq \mu_{X:j}^{2}\;for\;at\;least\;one\;j\in \left\{1,\ldots ,p-1\right\}.$$

In the following, we will introduce our proposed test specifically for hypothesis (2). We also investigate the theoretical properties of the test statistics.

### 4.2 The proposed MECAF test

Cao et al. (2018) proposed one maximum-type two-sample test for high-dimensional compositions by assuming that the covariance matrices of two groups are equal (see equation 9 of Cao et al. (2018)). In practice, it is unable to assess the assumption if $ma_{X}^{1}={\text{Σ}}_{X}^{2}$ or not. Thus, we consider a more general setting, where the equal covariance assumption is not required. For *j*∈{1,⋯,*p*} th component (taxon), let ${\bar{X}}_{j}^{1}=\frac{1}{n_{1}}\sum_{i=1}^{n_{1}}X_{ij}^{1}$ , and ${\bar{X}}_{j}^{2}=\frac{1}{n_{2}}\sum_{i=1}^{n_{2}}X_{ij}^{2}$ is the average of the CLR transformation of relative abundances. Our proposed test is given as follows:


$$T_{\text{MECAF}}=\underset{1\leq j\leq p-1}{\max\;}{\left(\sqrt{n_{1}+n_{2}}\frac{{\bar{X}}_{j}^{1}-{\bar{X}}_{j}^{2}}{\sqrt{{\hat{\sigma }}_{jj}}}\right)}^{2},$$

where ${\hat{\sigma }}_{jj}=\frac{n_{1}+n_{2}}{n_{1}}{\hat{\sigma }}_{X:jj}^{1}+\frac{n_{1}+n_{2}}{n_{2}}{\hat{\sigma }}_{X:jj}^{2}$ , and ${\hat{\sigma }}_{X:jj}^{g}=\frac{1}{n_{g}}\sum_{i=1}^{n_{g}}{\left(X_{ij}^{g}-{\bar{X}}_{j}^{g}\right)}^{2},g=1,2$ . We name it the MECAF test. As a maximum-type test statistic, it is in general better than sum-of-squares type statistics under sparse alternatives (Tony Cai et al. (2014)). The assumption of the equal high-dimensional covariance matrices for two groups is relaxed to allow for wider applicable conditions.

We successfully derived the asymptotic null distribution of *T*_MECAF_ given by


$$\text{Pr}\left({\left(2-\left(\log\;(p-1\right)\right)}^{-1}{)}^{-1}\left[T_{\text{MECAF}}-\left(h_{p}+\log\;4-\frac{log\;4}{2log\;(p-1)}\right)\right]<t\right)\to \exp\;(-\exp\;(-t)),$$

for any real number *t* as *n*_1_,*n*_2_,*p*→*∞* , where $h_{p}=2\log\;(p-1)-\left[\log\;(\log\;(p-1))+\log\;(4\pi )\right]+\frac{log\;(log\;(p-1))+log\;(4\pi )}{2log\;(p-1)}$ . Denote *q_α_* as the (1 - *α*) -quantile of the derived distribution function exp(-exp(-*t*)). Namely, *q_α_* = -log [log (1-*α*)^-1^]. We can define an asymptotic *α*-level test denoted by


$${\text{Φ}}_{1:\alpha }=\text{I}\left(T_{\text{MECAF}}\geq \left(2-\frac{1}{log\;(p-1)}\right)q_{\alpha }+h_{p}+\log\;4-\frac{log\;4}{2log\;(p-1)}\right).$$

The null hypothesis $H_{0}^{\left(2\right)}$ is rejected whenever Φ_1:_ *_α_* = 1. We also prove that the power of test Pr(Φ_1:_ *_α_* = 1) converges to 1 under some settings and $H_{1}^{\left(2\right)}$ as *n*_1_,*n*_2_,*p*→*∞* . All the detailed proof is given in the **Supplementary Material**.

## Data availability statement

The metagenomics abundance data of the CDI study is readily available through the R package “curatedMetagenomicsData”(Version 1.16.1) from the Bioconductor (https://bioconductor.org/packages/release/data/experiment/html/curatedMetagenomicData.html). The 16S rRNA amplicon sequencing data from the murine T1D study is publicly available at EBI with accession number ERP016357. 

## Ethics statement

No ethics approval or consent to participate was required for this study.

## Author contributions

ZL developed the proposed method and performed theoretical proof and simulation studies and manuscript writing. XY performed simulation and real data analyses and manuscript writing. HG performed theoretical proof and manuscript writing. TL performed simulation analyses. JH conceptualized the ideas for the proposed method, simulations, and real data analyses and performed manuscript writing. All authors contributed to the article and approved the submitted version.

## Funding

HG is funded by the Young Talents Project of Scientific Research Plan of the Hubei Provincial Department of Education (Grant No. Q20212506). JH is partly supported by NIH National Institute on Minority Health and Health Disparities under Award Number U54MD000538, and NIH National Institute on Aging under Award Number R33AG057382.

## Acknowledgments

The authors would like to thank the reviewers and editors for their valuable comments and suggestions.

## Conflict of interest

The authors declare that the research was conducted in the absence of any commercial or financial relationships that could be construed as a potential conflict of interest.

## Publisher’s note

All claims expressed in this article are solely those of the authors and do not necessarily represent those of their affiliated organizations, or those of the publisher, the editors and the reviewers. Any product that may be evaluated in this article, or claim that may be made by its manufacturer, is not guaranteed or endorsed by the publisher.
