# Supplementary material for: A maximum-type microbial differential abundance test with application to high-dimensional microbiome data analyses
Source: Front Cell Infect Microbiol. 2022 Oct 28;12:988717. doi: 10.3389/fcimb.2022.988717 (PMC9650337; doi:10.3389/fcimb.2022.988717)
Supplement: Supplementary file 1 [file DataSheet_1.pdf]

Supplementary Material for "A maximum-type multivariate differential abundance test with application to high-dimensional microbiome data analyses"

In Supp. Section 1, we summarize the theoretical properties of the proposed test MECAF. The following sections we detail the proof of the properties through propositions, corollaries, lemmas, and theorems. Specifically, in Supp. Section 2 we presents some useful lemmas and the proof of **Theorem 1**, **Proposition 1**, **Corollary 1**. Supp. Section 3 presents lemmas and the proof of **Lemma 1**. Supp. Section 4 presents the proofs of **Proposition 2**, **Proposition 3**, **Proposition 4**, **Lemma 2**, and **Theorem 2**. Supp. Section 5 presents detailed proof of **Theorem 3**.

S. 1 THEORETICAL PROPERTIES OF THE MECAF TEST STATISTICS

Denote $n = n_1 + n_2$, $m = p - 1$, and

$$\tilde{T}_1 = \max_{1 \leq j \leq m} \left(\sqrt{n_1 + n_2} \frac{\bar{X}_j^1 - \bar{X}_j^2}{\sqrt{\sigma_{jj}}} \right)^2,$$

where $\sigma_{jj} = \frac{n_1+n_2}{n_1}\sigma^1_{X:jj} + \frac{n_1+n_2}{n_2}\sigma^2_{X:jj}(j = 1, \cdots, m)$. Obviously, we obtain $\tilde{T}_1$ by replacing the estimator $\widehat{\sigma}_{jj}$ in T_{MECAF} with the real value σ_{jj}. We will derive asymptotic conclusion about $\tilde{T}_1$ at first.

Denote $\Sigma = (\sigma_{kj})_{k,j=1,\cdots,m} = \frac{n_1+n_2}{n_1}\Sigma^1_X + \frac{n_1+n_2}{n_2}\Sigma^2_X = (\frac{n_1+n_2}{n_1}\sigma^1_{X:kj} + \frac{n_1+n_2}{n_2}\sigma^2_{X:kj})_{k,j=1,\cdots,m}$, and $\Sigma_L = (\sigma_{L:kj})_{k,j=1,\cdots,m} = \frac{n_1+n_2}{n_1}\Sigma^1_L + \frac{n_1+n_2}{n_2}\Sigma^2_L = (\frac{n_1+n_2}{n_1}\sigma^1_{L:kj} + \frac{n_1+n_2}{n_2}\sigma^2_{L:kj})_{k,j=1,\cdots,m}$. Denote $\Xi = (r_{kj})_{k,j=1,\cdots,m}$, and $\Xi_L =$

$(r_{L:kj})_{k,j=1,\cdots,m}$, where

$$r_{kj} = \frac{\frac{n_1+n_2}{n_1}\sigma^1_{X:kj} + \frac{n_1+n_2}{n_2}\sigma^2_{X:kj}}{\sqrt{\frac{n_1+n_2}{n_1}\sigma^1_{X:kk} + \frac{n_1+n_2}{n_2}\sigma^2_{X:kk}}\sqrt{\frac{n_1+n_2}{n_1}\sigma^1_{X:jj} + \frac{n_1+n_2}{n_2}\sigma^2_{X:jj}}} \quad (k, j = 1, \cdots, m),$$

and

$$r_{L:kj} = \frac{\frac{n_1+n_2}{n_1}\sigma^1_{L:kj} + \frac{n_1+n_2}{n_2}\sigma^2_{L:kj}}{\sqrt{\frac{n_1+n_2}{n_1}\sigma^1_{L:kk} + \frac{n_1+n_2}{n_2}\sigma^2_{L:kk}}\sqrt{\frac{n_1+n_2}{n_1}\sigma^1_{L:jj} + \frac{n_1+n_2}{n_2}\sigma^2_{L:jj}}} \quad (k, j = 1, \cdots, m).$$

Let $S = (S_1, \cdots, S_m)^\top$, where $S_j = \frac{\sqrt{n_1+n_2}}{\sqrt{\sigma_{jj}}}(\bar{X}^1_j - \bar{X}^2_j) - (\mu^1_{X:j} - \mu^2_{X:j}) \quad (j = 1, \cdots, m)$. Obviously, the mean and covariance matrix of S are $(0, \cdots, 0)^\top$ and Ξ, respectively. Let $G_{X^g:i} = (G_{X^g:i1}, \ldots, G_{X^g:im})^\top (i = 1, \ldots, n_g)$ be independent samples with same Gaussian distribution with mean vector $(\mu^g_{X:1}, \ldots, \mu^g_{X:m})^T$ and covariance matrix Σ^g_X For $j = 1, \cdots, m$ and g=1,2, we calculate

$$G_j = \frac{\sqrt{n_1+n_2}}{n_1}\sum_{i=1}^{n_1}\frac{G_{X^1:ij} - \mu^1_{X:j}}{\sqrt{\sigma_{jj}}} - \frac{\sqrt{n_1+n_2}}{n_2}\sum_{i=1}^{n_2}\frac{G_{X^2:ij} - \mu^2_{X:j}}{\sqrt{\sigma_{jj}}}.$$

Then $G = (G_1, \ldots, G_m)^T$ is a Gaussian distributed random vector with mean $(0, \cdots, 0)^\top$ and covariance matrix Ξ.

In order to derive the asymptotic distributions of T_{MECAF} , we follow the following steps.

Step 1: Illustrate

$$P(\tilde{T}_1 > t) = P\left(\max_{1\le j\le m}(S_j)^2 > t\right) \approx P\Big(\max_{1\le j\le m}(G_j)^2 > t\Big),$$

under some regular conditions as $n_1, n_2, m \to \infty$.

Step 2: Show

$$P\left(\max_{1\le j\le m}(G_j)^2 > \Big(2 - (\log m)^{-1}\Big)t + c_m + \log 4 - \frac{\log 4}{2\log m}\right) \approx 1 - \exp\Big(-\exp(-t)\Big),$$

under $H_0^{(4)}$ and some regular conditions as $m \to \infty$, where $c_m = 2\log m - \big[\log\big(\log m\big) + \log(4\pi)\big] + \frac{\log(\log m)+\log(4\pi)}{2\log m}$.

Step 3: Illustrate

$$P\Big(T_{MECAF}>\Big(2-(\log m)^{-1}\Big)t+c_m+\log 4-\tfrac{\log 4}{2\log m}\Big)\approx P\Big(\tilde{T}_1>\Big(2-(\log m)^{-1}\Big)t+c_m+\log 4-\tfrac{\log 4}{2\log m}\Big),$$

under some regular conditions as $n_1, n_2, m \to \infty$.

Step 4: Illustrate the testing powers about T_{MECAF} converge to 1 under $H_1^{(4)}$ for regular settings and some regular conditions as $n_1, n_2, m \to \infty$.

We will present some theorems, corollaries and lemmas about **Step 1** in Supp. section 2; we will present lemmas about **Step 2** in Supp. section 3; we will present some theorems, corollaries and lemmas about **Step 3** in Supp. section 4; we will present some theorems, corollaries and lemmas about **Step 4** in Supp. section 5.

S. 2 HIGH-DIMENSIONAL GAUSSIAN APPROXIMATIONS

In order to illustrate **Step 1**, we need to obtain the high-dimensional Gaussian approximation theorems. Similar as Chernozhukov et al. (2013, 2017), we suppose some regular conditions first. Let constants $b_1, b_2, b_3, b_4, q > 0$, and $B_n \to \infty$, as $n \to \infty$, and impose the following regular conditions.

- Condition A1: $b_1 < \frac{n_1}{n_2} < b_2$.
- Condition A2: $\dfrac{\sigma^g_{X:jj}}{\sigma_{jj}} > b_3 \quad (j = 1, \cdots, m; g = 1, 2)$.
- Condition A3: $E\left[\left|\dfrac{X^g_{ij} - \mu^g_{X:j}}{\sqrt{\sigma_{jj}}}\right|^{2+a}\right] \le B_n^a \quad (i = 1, \ldots, n_g; j = 1, \cdots, m; a = 1, 2; g = 1, 2)$.
- Condition A4: $E\left[\exp\left\{\dfrac{|X^g_{ij} - \mu^g_{X:j}|}{\sqrt{\sigma_{jj}}B_n}\right\}\right] \le 2 \quad (i = 1, \ldots, n_g; j = 1, \cdots, m; g = 1, 2$).

- Condition A5: $E\left[\left(\max_{1\le j\le m}\frac{|X_{ij}^g-\mu_{X:j}^g|}{\sqrt{\sigma_{jj}}B_n}\right)^q\right]\le 2\ (i=1,\dots,n_g; j=1,\cdots,m; g=1,2)$.

Remark Similar regular conditions A2-A5 for central limit theorems in one population are also imposed in Chernozhukov et al. (2017). Condition A1 is essential. Condition A2 is somewhat weak. Condition A3-A4 is stronger than Condition A2.

With these regular conditions, we define some notations which are useful in the high-dimensional Gaussian approximation theorems. Define

$$L_n=\max_{1\le j\le m}\frac{(n_1+n_2)^2}{\sqrt{\sigma_{jj}^3}}\left\{\frac{E[|X_{ij}^1-\mu_{X:j}^1|^3]}{n_1^2}+\frac{E[|X_{ij}^2-\mu_{X:j}^2|^3]}{n_2^2}\right\}.$$

As $\phi\ge 1$, define

$$M_{n,X^1,X^2}(\phi)=\left(\frac{n_1+n_2}{n_1}\right)^2E[\max_{1\le j\le m}\left(\frac{|X_{ij}^1-\mu_{X:j}^1|}{\sqrt{\sigma_{jj}}}\right)^3I\{\max_{1\le j\le m}|X_{ij}-\mu_{X:j}^1|>\frac{\sqrt{\sigma_{jj}}n_1}{4\phi\sqrt{n}\log m}\}]$$

$$+\left(\frac{n_1+n_2}{n_2}\right)^2E[\max_{1\le j\le m}\left(\frac{|X_{ij}^2-\mu_{X:j}^2|}{\sqrt{\sigma_{jj}}}\right)^3I\{\max_{1\le j\le m}|X_{ij}^2-\mu_{X:j}^2|>\frac{\sqrt{\sigma_{jj}}n_2}{4\phi\sqrt{n}\log m}\}].$$

Similarly, define

$$M_{n,G}(\phi)=\left(\frac{n_1+n_2}{n_1}\right)^2E[\max_{1\le j\le m}\left(\frac{|G_{X^1:ij}-\mu_{X:j}^1|}{\sqrt{\sigma_{jj}}}\right)^3I\{\max_{1\le j\le m}|G_{X^1:ij}-\mu_{X:j}^1|>\frac{\sqrt{\sigma_{jj}}n_1}{4\phi\sqrt{n}\log m}\}]$$

$$+\left(\frac{n_1+n_2}{n_2}\right)^2E[\max_{1\le j\le m}\left(\frac{|G_{X^2:ij}-\mu_{X:j}^2|}{\sqrt{\sigma_{jj}}}\right)^3I\{\max_{1\le j\le m}|G_{X^2:ij}-\mu_{X:j}^2|>\frac{\sqrt{\sigma_{jj}}n_2}{4\phi\sqrt{n}\log m}\}].$$

Let $\mathcal{A}^{re}$ be the class of all hyperrectangles in $\mathbb{R}^m$, that is, $\mathcal{A}^{re}$ consists of all sets A of the form

$$A=\{(\xi_1,\cdots,\xi_m)^\top\in\mathbb{R}^m: a_j<\xi_j<f_j(-\infty\le a_j\le f_j\le+\infty; j=1,\dots,m)\}.$$

With these notations and conditions, we can obtain the following asymptotic theorems.

Theorem 1 Under Condition A1 and A2, there exist constant $K_1, K_2 > 0$, such that for every constant $\bar{L}_n \geq L_n$, we have

$$\sup_{A \in \mathcal{A}^{re}} \left| P(S \in A) - P(G \in A) \right| \leq K_1 \left[\left(\frac{\bar{L}_n^2 \log^7 m}{n} \right)^{\frac{1}{6}} + \frac{M_{n,X^1,X^2}(\phi_n) + M_{n,G}(\phi_n)}{\bar{L}_n} \right],$$

where $\phi_n := K_2 \left(\frac{\bar{L}_n^2 \log^4 m}{n} \right)^{-\frac{1}{6}}$, and the constant K_1 depends only on b_3.

Remark: The bound in **Theorem 1** is similar as that of Chernozhukov et al. (2017).

When conditions are strengthened, we can obtain the following proposition.

Proposition 1 Suppose that Condition A1, A2 and A3 are satisfied. Then under Condition A4, we can obtain that

$$\sup_{A \in \mathcal{A}^{re}} \left| P(S \in A) - P(G \in A) \right| \leq C_1 \left(\frac{B_n^2 \log^7(mn)}{n} \right)^{\frac{1}{6}},$$

where the constant C_1 depends only on b_3; while under Condition A5, we can obtain that

$$\sup_{A \in \mathcal{A}^{re}} \left| P(S \in A) - P(G \in A) \right| \leq C_2 \left\{ \left(\frac{B_n^2 \log^7(mn)}{n} \right)^{\frac{1}{6}} + \left(\frac{B_n^2 \log^3(mn)}{n^{1-\frac{2}{q}}} \right)^{\frac{1}{3}} \right\},$$

where the constant C_2 depends only on b_3 and q.

Based on **Proposition 1** , we can obtain the following corollary.

Corollary 1 Under Condition A1, A2, and A3, we can obtain that

$$\sup_{t\in\mathbb{R}} |P(\max_{1\le j\le m} S_j^2 < t) - P(\max_{1\le j\le m} G_j^2 < t)| \le C_1 \left(\frac{B_n^2 \log^7(mn)}{n}\right)^{\frac{1}{6}},$$

where the constant C_1 depends only on b_3.

Remark: Under Condition A1, A2 and A3, we can obtain that the bound $C_1 \left(\frac{B_n^2 \log^7(mn)}{n}\right)^{\frac{1}{6}} = o(1)$, as $n_1, n_2, m \to \infty$ and $\log m = o(n^{1/7})$. So $P(\tilde{T}_1 > t) = P\left(\max_{1\le j\le m} (S_j)^2 > t\right) \approx P\Big(\max_{1\le j\le m} (G_j)^2 > t\Big)$, under some regular conditions as $n_1, n_2, m \to \infty$. This completes **Step 1**.

All detailed proofs of **Theorem 1**,**Proposition 1**, and **Corollary 1** are presented in Appendix A.

S. 3 ASYMPTOTIC DISTRIBUTIONS OF EXTREME STATISTICS FROM HIGH-DIMENSIONAL GAUSSIAN RANDOM VECTOR

In order to illustrate **Step 2**, we list on condition. Denote $c_m = 2\log m - \big[\log\big(\log m\big) + \log(4\pi)\big] + \frac{\log(\log m)+\log(4\pi)}{2\log m}$. Suppose the following condition is satisfied.

- Condition B1: For any real number t, $\sum_{1\le i<j\le m} \frac{|r_{ij}|}{\sqrt{1-r_{ij}^2}} \exp\left\{-\frac{\left[2-(\log m)^{-1}\right]t+c_m}{1+|r_{ij}|}\right\} \to 0$, as $m\to\infty$.

According to the extreme theory about Gaussian distribution (Leadbetter et al. (2012); Li et al. (2021)), we have the following lemmas.

Lemma 1 Let $G = (G_1, \cdots, G_m)^\top$ follow m-dimension multivariate normal distribution, with m dimension mean vector $\mu = (0, \cdots, 0)^\top$, covariance matrix Ξ. Under Condition B1,

we have the following conclusion. $\forall t \in \mathbb{R}$,

$$\lim_{m\to\infty} P\left(\frac{[\max_{s=1,\cdots,m}(|G_j|)]^2 - \left(c_m + \log 4 - \frac{\log 4}{2\log m}\right)}{2-(\log m)^{-1}} < t\right) = \exp\left(-\exp(-t)\right).$$

Remark: We have showed $P\left(\max_{1\le j\le m}(G_j)^2 > \left(2-(\log m)^{-1}\right)t + c_m + \log 4 - \frac{\log 4}{2\log m}\right)$ $\approx 1 - \exp\Big(-\exp(-t)\Big)$, under $H_0^{(4)}$ and Condition B1 as $m \to \infty$ according to **Lemma 1**. This completes **Step 2**.

Detailed proof of **Lemma 1** is presented in Appendix B.

S. 4 ASYMPTOTIC RESULTS OF THE NOVEL TEST UNDER NULL HYPOTHESIS

To develop the asymptotic distributions of T_1 under null hypothesis, we need to derive the asymptotic distributions of $\tilde{T}_1$ under null hypothesis at first. In order to illustrate **Step 3**, we list six conditions.

Denote the correlation matrices of L_1^g and X_1^g by $R_{L^g} = (\rho_{L^g:kj})$ and $R_{X^g} = (\rho_{X^g:kj})$ (g=1,2) . Define $\sigma_{L:kj} = \dfrac{n_1+n_2}{n_1}\sigma^1_{L:kj} + \dfrac{n_1+n_2}{n_2}\sigma^2_{L:kj}$, $\rho_{L:kj} = \dfrac{\sigma_{L:kj}}{\sqrt{\sigma_{L:kk}\sigma_{L:jj}}}$, $\sigma_{L:k\cdot} = \frac{1}{m}\sum_{j=1}^{m}\sigma_{L:kj}$, $\sigma_{L:\cdot j} = \frac{1}{m}\sum_{k=1}^{m}\sigma_{L:kj}$, $\sigma_{L:\cdot\cdot} = \frac{1}{m^2}\sum_{k=1}^{m}\sum_{j=1}^{m}\sigma_{L:kj}$.

We impose the following conditions on the covariance structures of the log basis variables.

- Condition C1: $\dfrac{1}{b_4} \le \sigma^g_{L:jj} \le b_4$ for $j = 1, \cdots, m; g = 1, 2$ and some constants $b_4 > 0$;
- Condition C2: $\max_{1\le k,j\le m}|\rho_{L^g:kj}| \le b_5$ for g=1,2 and some constants $1 > b_5 > 0$;
- Condition C3: $\max_{1\le j\le m}\sum_{k=1}^{m}(\rho_{L^g:kj})^2 \le b_6$ for g=1,2 and some constants $b_6 > 0$;

- Condition C4: $E\left[\exp\left\{\frac{b_7(L^g_{1j}-\mu^g_{L:j})^2}{\sigma^g_{L:jj}}\right\}\right] \leq b_8$ for $j = 1, \cdots, m; g = 1, 2$ and some constants $b_7, b_8 > 0$;;
- Condition C5: $\log m = \circ(n^{\frac{1}{3}})$;
- Condition C6: For any real number t, $\sum_{1\leq i<j\leq m} \frac{|r_{L:ij}|}{\sqrt{1-r^2_{L:ij}}} \exp\left\{-\frac{\left[2-(\log m)^{-1}\right]t+c_m}{1+|r_{L:ij}|}\right\} \to$ 0, as $m \to \infty$.

Condition C1-C3 are mild and standard in the high-dimentional testing literature. Condition C1 requires that the variances be bounded away from zero and infinity. Condition 2 is mild since $\max_{1\leq i<j\leq m} |\rho_{L^g:kj}| = 1$ would imply that Σ_{L^g} (g=1,2) are singular.

Under conditions Condition C1 and C3, the following propositions show that the similar properties are satisfied by Σ_{X^g} (g=1,2).

Proposition 2 Assume that Condition C1 and C3 hold. For sufficiently large m and some constant $b_9 > 0$, we can obtain that

$$\frac{1}{b_9} \leq \sigma^g_{X:jj} \leq b_9(j = 1, \cdots, m; g = 1, 2).$$

Proposition 3 Under Condition C1 and C3, we can obtain that

$$\max_j \frac{|\hat{\sigma}_{jj} - \sigma_{jj}|}{\sigma_{jj}} = O_p\left(\sqrt{\frac{\log m}{n}}\right).$$

Proposition 4 Under Condition C1 and C6 , we can obtain , for any real number t,

$$\sum_{1\leq i<j\leq m} \frac{|r_{ij}|}{\sqrt{1-r^2_{ij}}} \exp\left\{-\frac{\left[2-(\log m)^{-1}\right]t+c_m}{1+|r_{ij}|}\right\} \to 0, as\ m \to \infty.$$

Based on the high dimensional Gaussian approximation results and asymptotic distributions about extremes from high-dimensional Gaussian random vector, one can derive the asymptotic result about $\tilde{T}_1$ under null hypothesis, which is presented in the following lemmas.

Lemma 2 Under Condition A1, A2, A3 and C6 as $H_0^{(4)}$ in (2.1) holds, for any real number t,

$$\lim_{n_1,n_2,m\to\infty} P\left(\frac{\tilde{T}_1-\left(c_m+\log 4-\frac{\log 4}{2\log m}\right)}{2-(\log m)^{-1}}<t\right)=\exp(-\exp(-t)). \tag{S1}$$

Based on **Proposition 2** , **Proposition 3** , **Proposition 4** , **Lemma 1** , **Lemma 2** , we can obtain the asymptotic results about T_{MECAF} under null hypothesis, which are presented in the following theorem.

Theorem 2 Under Condition A1, A2, A3, C1, C3, C5 and C6, as $H_0^{(4)}$ in (2.1) holds, for any real number t,

$$\lim_{n_1,n_2,m\to\infty} P\left(\frac{T_{MECAF}-\left(c_m+\log 4-\frac{\log 4}{2\log m}\right)}{2-(\log m)^{-1}}<t\right)=\exp(-\exp(-t)). \tag{S2}$$

Remark: According to **Lemma 2**, we have showed

$$P\left(\tilde{T}_{MECAF}>\left(2-(\log m)^{-1}\right)t+c_m+\log 4-\frac{\log 4}{2\log m}\right)\approx 1-\exp\Big(-\exp(-t)\Big),$$

under $H_0^{(4)}$ and some regular conditions as $m\to\infty$. According to **Theorem 2**, we have showed

$$P\left(T_{MECAF}>\left(2-(\log m)^{-1}\right)t+c_m+\log 4-\frac{\log 4}{2\log m}\right)\approx 1-\exp\Big(-\exp(-t)\Big),$$

under $H_0^{(4)}$ and some regular conditions as $m \to \infty$. Then we can obtain that

$$P\left(T_1 > \left(2-(\log m)^{-1}\right)t + c_m + \log 4 - \frac{\log 4}{2\log m}\right) \approx P\left(\tilde{T}_1 > \left(2-(\log m)^{-1}\right)t + c_m + \log 4 - \frac{\log 4}{2\log m}\right),$$

under some regular conditions as $n_1, n_2, m \to \infty$. So we accomplish **Step 3**.

All detailed proofs of **Proposition 2**, **Proposition 3**, **Proposition 4**, **Lemma 2**, and **Theorem 2** are presented in Appendix C.

S. 5 ASYMPTOTIC POWERS OF TWO NOVEL TESTS UNDER ALTERNATIVE HYPOTHESIS

To study the asymptotic power of the novel test. We consider the alternative

$$H_1^{(5)} : \mu_{L:j}^1 \neq \mu_{L:j}^2 + c, j \in \xi; \mu_{L:j}^1 = \mu_{L:j}^2 + c, j \in \xi^c \tag{S3}$$

for some $c \in \mathbb{R}$ and $\xi \subset \{1, \cdots, m\}$ with cardinality ξ, where ξ^c denotes the complement of ξ. Without loss of generality, define the signal vector $\delta = \{\delta_1, \ldots, \delta_m\}^T$ by

$$\mu_{L:j}^1 - \mu_{L:j}^2 - c = \delta_j \sqrt{\sigma_{L:jj}} \sqrt{\frac{\log m}{n}} (j = 1, \cdots, m); \tag{S4}$$

under $H_1^{(5)}$, and we have $\delta_j \neq 0$ if and only if $j \in \xi$. Summing the equations and rearranging, we can obtain

$$c = \bar{\mu}_L^1 - \bar{\mu}_L^2 - \frac{1}{m}\sum_{j=1}^{m} \delta_j \sqrt{\sigma_{L:jj}} \sqrt{\frac{\log m}{n}} = \bar{\mu}_L^1 - \bar{\mu}_L^2 - O\left(\frac{\| \delta \|_1}{m}\sqrt{\frac{\log m}{n}}\right), \tag{S5}$$

where $\bar{\mu}_{L^g} = \frac{1}{m}\sum_{j=1}^{m} \mu_{L:j}^g$ (g=1,2), $\| \delta \|_1 = \sum_{j=1}^{m} |\delta_j|$, and we have used the fact that $\lim_j \sigma_{L:jj}$=O(1) by Condition C1. Since $X_1^g = UX_1^g$, we can get $\mu_{X:j}^g = \mu_{L:j}^g - \bar{\mu}_{L^g}$

(g=1,2), and we see that $H_1^{(5)}$ implies

$$\begin{aligned} \mu_{X:j}^1 - \mu_{X:j}^2 &= \left[\delta_j \sqrt{\sigma_{L:jj}} + O\left(\frac{\parallel \delta \parallel_1}{m}\right)\right] \sqrt{\frac{\log m}{n}}, j \in \xi, \\ \mu_{X:j}^1 - \mu_{X:j}^2 &= O\left(\frac{\parallel \delta \parallel_1}{m} \sqrt{\frac{\log m}{n}}\right), j \in \xi^c. \end{aligned} \tag{S6}$$

The following theorem presents asymptotic power about the novel test.

Theorem 3 Under Condition A1, A2, C1, C3, C4, C5, and C6, as $H_1^{(5)}$ holds, if $\parallel \delta \parallel_1 = o(m)$ and $\lim_{j \in \xi} |\delta_j| \geq \sqrt{2} + \varepsilon$ for some constant $\varepsilon > 0$, then

$$\lim_{n_1, n_2, m \to \infty} P\left(\Phi_{1:\alpha} = 1\right) = 1. \tag{S7}$$

Remark According to Theorem 3 , we have showed that the testing powers about T_1 converge to 1 under $H_1^{(4)}$ for regular settings and some regular conditions as $n_1, n_2, m \to \infty$. So we accomplish **Step 4**.

Detailed proof of **Theorem 3** are presented in Appendix D.

APPENDIX A

Appendix A presents some useful lemmas and the detailed proofs of **Theorem 1**,**Proposition 1**,**Corollary 1** in Supp. Section 2.

Lemma A.1 Let Y=$(Y_1 \ldots Y_m)^T$ be a centered Gaussian random vector in $\mathbb{R}^m$ such that $E[Y_j^2] \geq b$ for all $j = 1, \ldots, m$ and some constants $b > 0$. Then for every $y \in \mathbb{R}^m$ and $a > 0$,

$$P(Y \leq y + a) - P(Y \leq y) \leq Ca\sqrt{\log m},$$

where the constant C depends only on b.

Proof Please refer to Nazarov (2003).

Lemma A.2 Define an undecreasing function Φ_i:$\mathbb{R} \mapsto [0, \infty)$ (i=1,2) and independent real valued random variables ξ_i (i=1,2), then we can get

$$E[\Phi_1(\xi_1)\Phi_2(\xi_2)] \leq E[\Phi_1(\xi_1)\Phi_2(\xi_1)] + E[\Phi_1(\xi_2)\Phi_2(\xi_2)].$$

Proof Because $\Phi_i(\xi_i) \geq 0$, we can get $\Phi_2(\xi_1)\Phi_1(\xi_2) \geq 0$. Therefore, $E[\Phi_1(\xi_1)\Phi_2(\xi_2)] \leq E[\Phi_1(\xi_1)\Phi_2(\xi_2)] + E[\Phi_2(\xi_1)\Phi_1(\xi_2)]$. Φ_i is undecreasing, so $(\Phi_1(\xi_1) - \Phi_1(\xi_2)) \times (\Phi_2(\xi_1) - \Phi_2(\xi_2)) \geq 0$, and $E[(\Phi_1(\xi_1) - \Phi_1(\xi_2))(\Phi_2(\xi_1) - \Phi_2(\xi_2))] \geq 0$, namely $E[\Phi_1(\xi_1)\Phi_2(\xi_1)] + E[\Phi_1(\xi_2)\Phi_2(\xi_2)] \geq E[\Phi_1(\xi_1)\Phi_2(\xi_2)] + E[\Phi_2(\xi_1)\Phi_1(\xi_2)]$. Therefore, $E[\Phi_1(\xi_1)\Phi_2(\xi_2)] \leq E[\Phi_1(\xi_1)\Phi_2(\xi_1)] + E[\Phi_1(\xi_2)\Phi_2(\xi_2)]$.

Lemma A.3 Let ς be a non-negative random variable such that $P(\varsigma > x) \leq A\exp\{-\frac{x}{B}\}$ for all $x \geq 0$ and for some constants A,B ¿ 0. Then for every $t \geq 0$, $E[\varsigma^3 I(\varsigma > t)] \leq 6A(t + B)^3 \exp\{-\frac{t}{B}\}$.

Proof Observe that

$$E[\varsigma^3 1(\varsigma > t)] = 3\int_0^t P(\varsigma > t)x^2 dx + 3\int_t^\infty P(\varsigma > t)x^2 dx = P(\varsigma > t)t^3 + 3\int_t^\infty P(\varsigma > t)x^2 dx.$$

Since $P(\varsigma > x) \le A\exp\{-\frac{x}{B}\}$, using integration by parts, we have

$$\int_t^\infty P(\varsigma > t)x^2 dx \le A(Bt^2 + 2B^2 t + 2B^3)\exp\{-\frac{t}{B}\},$$

which leads to

$$E[\varsigma^3 1(\varsigma > t)] \le A(t^3 + 3Bt^2 + 6B^2 t + 6B^3)\exp\{-\frac{t}{B}\} \le 6A(t+B)^3\exp\{-\frac{t}{B}\}.$$

Lemma A.4 For any $1 \le j, k, l \le m$, define

- $\partial_j F_\beta(z) = \dfrac{\exp\{\beta z_j\}}{\sum_{j=1}^m \exp\{\beta z_j\}} := \pi_j(z),$
- $\partial_{j,k} F_\beta(z) = \beta(\pi_j\delta_{kj} - \pi_j\pi_k)(z) := \beta w_{jk}(z),$
- $\partial_{j,k,l} F_\beta(z) = \beta^2(\pi_j\delta_{jl}\delta_{jk} - \pi_j\pi_l\delta_{jk} - \pi_j\pi_k(\delta_{jl} + \delta_{kl}) + 2\pi_j\pi_k\pi_l)(z) := \beta^2 q_{jkl}(z),$

where $\delta_{jk} = I\{j = k\}$, $\delta_{jl} = I\{j = l\}$, $\delta_{kl} = I\{k = l\}$. We can obtain that $\pi_j(z) \ge 0$, $\sum_{j=1}^m \pi_j(z) = 1$, $\sum_{j,k=1}^m |w_{jk}(z)| \le 2$, $\sum_{j,k,l=1}^m |q_{jkl}(z)| \le 6$.

Proof Observe that

$$\sum_{j=1}^m \pi_j(z) = \sum_{j=1}^m \left(\frac{\exp\{\beta z_j\}}{\sum_{j=1}^m \exp\{\beta z_j\}} \right) = 1.$$

$$\sum_{j,k=1}^m |w_{jk}(z)| \le \sum_{j=1}^m \pi_j(z) + (\sum_{j=1}^m \pi_j(z))^2 \le 2.$$

$$\sum_{j,k,l=1}^{m} |q_{jkl}(z)| \leq \sum_{j,k,l=1}^{m} \pi_j \delta_{jl} \delta_{jk}(z) + 3 \sum_{j,k,l=1}^{m} \pi_j \pi_l \delta_{jk}(z) + 2 \sum_{j,k,l=1}^{m} \pi_j \pi_l \pi_k \leq 6.$$

Lemma A.5 For any $1 \leq j, k, l \leq m$, By the chain rule,

- $\partial_j f(z) = (\partial g(F_\beta)\pi_j)(z)$,
- $\partial_{j,k} f(z) = (\partial^2 g(F_\beta)\pi_j \pi_k + \partial g(F_\beta)\beta w_{jk})(z)$,
- $\partial_{j,k,l} f(z) = (\partial^3 g(F_\beta)\pi_j \pi_k \pi_l + \partial^2 g(F_\beta)\beta(w_{jk}\pi_l + w_{jl}\pi_k + w_{kl}\pi_j) + \partial g(F_\beta)\beta^2 q_{jkl})(z)$.

By the defination of function g(t),

$$\partial g(F_\beta) \leq \frac{1}{\phi^{-1} - 0} = \phi.$$

Define the upper bounds of the derivatives of f(z),

- $|\ \partial_{j,k} f(z)| \leq (\phi^2 \pi_j \pi_k + \phi\beta W_{jk})(z) := U_{jk}(z)$,
- $|\ \partial_{j,k,l} f(z)| \leq (\phi^3 \pi_j \pi_k \pi_l + \phi^2 \beta(W_{jk}\pi_l + W_{jl}\pi_k + W_{kl}\pi_j) + \phi\beta^2 Q_{jkl})(z) := U_{jkl}(z)$,

where $W_{jk}(z) := (\pi_j \delta_{kj} + \pi_j \pi_k)(z)$, $Q_{jkl}(z) := (\pi_j \delta_{jl} \delta_{jk} + \pi_j \pi_l \delta_{jk} + \pi_j \pi_k \delta_{jl} + \pi_j \pi_k \delta_{kl} + 2\pi_j \pi_k \pi_l)(z)$.

We can obtain that $\sum\limits_{j,k=1}^{m} U_{jk}(z) \leq (\phi^2 + 2\phi\beta)$, and $\sum\limits_{j,k,l=1}^{m} U_{jkl}(z) \leq (\phi^3 + 6\phi^2\beta + 6\phi\beta^2)$.

Proof Observe that

$$\begin{aligned} \sum_{j,k=1}^{m} U_{jk}(z) &= \phi^2 \sum_{j,k=1}^{m} \pi_j \pi_k(z) + \phi\beta \sum_{j,k=1}^{m} \pi_j \delta_{jk}(z) + \phi\beta \sum_{j,k=1}^{m} \pi_j \pi_k(z) \\ &\leq \phi^2 \left(\sum_{j=1}^{m} \pi_j(z) \right)^2 + \phi\beta \sum_{j=1}^{m} \pi_j(z) + \phi\beta \left(\sum_{j=1}^{m} \pi_j(z) \right)^2 . \end{aligned} \tag{S8}$$

By **Lemma A.4**, we know that $\sum\limits_{j=1}^{m} \pi_j(z) = 1$, so we can get

$$\sum_{j,k=1}^{m} U_{jk}(z) \le \phi^2 + 2\phi\beta.$$

$$\begin{aligned}
\sum_{j,k,l=1}^{m} U_{jkl}(z) &= \phi^3 \sum_{j,k,l=1}^{m} \pi_j\pi_k\pi_l(z) + 3\phi^2\beta \sum_{j,k,l=1}^{m} W_{jk}\pi_l(z) + \phi\beta^2 \sum_{j,k,l=1}^{m} Q_{jkl}(z) \\
&= \phi^3 \left(\sum_{j=1}^{m} \pi_j(z)\right)^3 + 3\phi^2\beta \sum_{j,l,k=1}^{m} \pi_j\pi_l\delta_{jk}(z) + 3\phi^2\beta \sum_{j,l,k=1}^{m} \pi_j\pi_k\pi_l(z) \\
&+ 4\phi\beta^2 \sum_{j,l,k=1}^{m} \pi_j\delta_{jl}\delta_{jk} + 2\phi\beta^2 \left(\sum_{j=1}^{m} \pi_j(z)\right)^3 \\
&\le \phi^3 + 6\phi^2\beta + 6\phi\beta^2.
\end{aligned} \tag{S9}$$

The last inequality works because $\sum\limits_{j=1}^{m} \pi_j(z) = 1$.

Lemma A.6 For any $z \in \mathbb{R}^m$ and $\omega \in \mathbb{R}^m$, if $\max\limits_{1\le j\le m} |\omega_j|\beta \le 1$, then for any $\tau \in [0,1]$ and any $0 \le j,k,l \le m$, we have

$$U_{jkl}(z) \lesssim U_{jkl}(z + \tau\omega) \lesssim U_{jkl}(z).$$

Proof

$$
\begin{aligned}
\pi_j(z+\tau\omega) &= \frac{\exp\{z_j\beta+\tau\omega_j\beta\}}{\sum\limits_{j=1}^{m}\exp\{z_j\beta+\tau\omega_j\beta\}} \\
&\leq \frac{\exp\{z_j\beta\}}{\sum\limits_{j=1}^{m}\exp\{z_j\beta\}} \times \frac{\exp\{\tau\max\limits_{1\leq j\leq m}|\omega_j|\beta\}}{\exp\{-\tau\max\limits_{1\leq j\leq m}|\omega_j|\beta\}} \\
&\leq \pi_j\exp\{2\tau\max_{1\leq j\leq m}|\omega_j|\beta\}.
\end{aligned} \tag{S10}
$$

Because $\beta\max\limits_{1\leq j\leq m}|\omega_j|\leq 1$, $\tau\leq 1$, we can get $\pi_j(z+\tau\omega)\leq e^2\pi_j$.

Similarly,

$$
\begin{aligned}
\pi_j(z+\tau\omega) &= \frac{\exp\{z_j\beta+\tau\omega_j\beta\}}{\sum\limits_{j=1}^{m}\exp\{z_j\beta+\tau\omega_j\beta\}} \\
&\geqslant \frac{\exp\{z_j\beta\}}{\sum\limits_{j=1}^{m}\exp\{z_j\beta\}} \times \frac{\exp\{-\tau\max\limits_{1\leq j\leq m}|\omega_j|\beta\}}{\exp\{\tau\max\limits_{1\leq j\leq m}|\omega_j|\beta\}} \\
&\geqslant e^{-2}\pi_j.
\end{aligned} \tag{S11}
$$

In conclusion,

$$\pi_j(z)\lesssim\pi_j(z+\tau\omega)\lesssim\pi_j(z).$$

Since U_{jkl} is a finite sum of $\pi_j,\pi_k,\pi_l,\delta_{jk}$, we can get

$$U_{jkl}(z)\lesssim U_{jkl}(z+\tau\omega)\lesssim U_{jkl}(z).$$

In order to prove **Theorem 1**, we denote

$$\Delta_1 = \sup_{A\in\mathcal{A}^{re}, v=1} \left|P(\sqrt{v}S + \sqrt{1-v}G \in A) - P(G \in A)\right|,$$

which is obviously less than

$$\rho_1 = \sup_{A\in\mathcal{A}^{re}, v\in[0,1]} \left|P(\sqrt{v}S + \sqrt{1-v}G \in A) - P(G \in A)\right|.$$

Note $W_i = (W_{i1}, \ldots, W_{im})$ is the copy of $G_i = (G_{i1}, \ldots, G_{im})$ $(i = 1, \ldots, n)$, and $W = \frac{1}{n}\sum_{i=1}^{n} W_n$. So,

$$\rho_1 = \sup_{A\in\mathcal{A}^{re}, v\in[0,1]} \left|P(\sqrt{v}S + \sqrt{1-v}G \in A) - P(W \in A)\right|.$$

We bound ρ_1 in the following **Lemma A.7** .

Lemma A.7 Under Condition A1 and A2, ρ_1 satisfies the following inequality for all $\phi \geq 1$:

$$\rho_1 \lesssim \frac{\phi^2(\log m)^2}{\sqrt{n}}\Big\{\phi L_n \rho_1 + L_n\sqrt{\log m} + \phi\Big[M_{n,X^1,X^2}(2\phi) + M_{n,G}(2\phi)\Big]\Big\} + \frac{\sqrt{\log m}}{\phi}.$$

up to a constant $K^{'}$ that depends only on b_3.

Remark Since the proof of **Lemma A.7** is important and complex, we divide the proof process into the following steps for easy understanding.

- **Step 1** : Simplify the problem of finding the upper bounding of ρ_1 to the problem of finding the upper bounding of $\sup_{y\in\mathbb{R}^m, v\in[0,1]} \left|P(\sqrt{v}S + \sqrt{1-v}G < y) - P(A < y)\right|$.
- **Step 2** : Transform the probability into the expectation of the indiative function.

- **Step 3** : By using **Lemma A.1** transform the problem to finding the expectation of the differentiable function of random vectors.
- **Step 4** : By using Taylor's theorem and Stein's leave-one-out method, divide the function into three parts and finding their upper bounds separately.

Proof Step 1 Define for any $i \in (1, \ldots, n)$, $\widetilde{Y}_i \in \mathbb{R}^{2m}$,$\widetilde{G}_i \in \mathbb{R}^{2m}$ satisfy:

- when $j = 1, \ldots, m$, $\widetilde{Y}_{ij} = Y_{ij}$, $\widetilde{G}_{ij} = G_{ij}$,
- when $j = m+1, \ldots, 2m$, $\widetilde{Y}_{ij} = -Y_{i,j-m}$, $\widetilde{G}_{ij} = -G_{i,j-m}$,

Note $\widetilde{S}_j = \dfrac{1}{\sqrt{n}} \sum\limits_{i=1}^{n} \widetilde{Y}_{ij}$ $(j = 1, \ldots, 2m)$, $y = (y_1, \ldots, y_{2m}) \in \mathbb{R}^{2m}$ Then we have:

$$\begin{aligned}
\sup_{y \in \mathbb{R}^{2m}} P(\widetilde{S} < y) &= \sup_{y \in \mathbb{R}^{2m}} P(\widetilde{S}_1 < y_1, \ldots, \widetilde{S}_m < y_m, \ldots, \widetilde{S}_{m+1} < y_{m+1}, \ldots, \widetilde{S}_{2m} < y_{2m}) \\
&= \sup_{y \in \mathbb{R}^{2m}} P(\widetilde{S}_1 < y_1, \ldots, \widetilde{S}_m < y_m, \ldots, -\widetilde{S}_{m+1} > -y_{m+1}, \ldots, -\widetilde{S}_{2m} > -y_{2m}) \\
&= \sup_{y \in \mathbb{R}^{2m}} P(\widetilde{S}_1 < y_1, \ldots, \widetilde{S}_m < y_m, \ldots, \widetilde{S}_1 > -y_{m+1}, \ldots, \widetilde{S}_m > -y_{2m}) \\
&= \sup_{y \in \mathbb{R}^{2m}} P(-y_{m+1} < \widetilde{S}_1 < y_1, \ldots, -y_{2m} < \widetilde{S}_m < y_m) \\
&= \sup_{A \in \mathcal{A}^{re}} P(\widetilde{S} \in A)
\end{aligned} \tag{S12}$$

As a result, in order to bound ρ_1, write

$$\rho_2 = \sup_{y \in \mathbb{R}^m, v \in [0,1]} \left| P(\sqrt{v} S + \sqrt{1-v} G < y) - P(W < y) \right|.$$

Step 2 Clearly, for any $X \in \mathbb{R}^m$, $P(X \leq y) = E[I(X \leq y)] = E[I(\max\limits_{1 \leq j \leq m} (X_j - y_j) \leq 0)]$. However, neither the function

$$\omega \to \max_{1 \leq j \leq m} (\omega_j - y_j),$$

nor the indicative function is derivable. In order to complete the proof, we replace those two functions by smooth functions: Let $\beta = \phi \log m$, define

$$F_\beta(\omega) := \frac{\log(\sum_{j=1}^{m} \exp\{\beta(\omega_j - y_j)\})}{\beta}, \quad \omega \in \mathbb{R}^m,$$

$$\begin{aligned} F_\beta(\omega) &\leq \frac{\log(m \exp\{\beta \max_{1\leq j\leq m}(\omega_j - y_j)\})}{\beta} \\ &= \frac{\log m}{\beta} + \max_{1\leq j\leq m}(\omega_j - y_j) \end{aligned} \tag{S13}$$

Therefore, $F_\beta(\omega)$ satisfies the following property :

$$0 \leq F_\beta(\omega) - \max_{1\leq j\leq m}(\omega_j - y_j) \leq \frac{\log m}{\beta} = \phi^{-1}.$$

Define a third order differentiable function whose derivative is bounded:

$$g_0(t) = \begin{cases} 1 & t \leq 0 \\ a(t) \in (0,1) & 0 < t < 1 \\ 0 & t \geq 1. \end{cases}$$

Let g(t)=$g_0(\phi t)$:

$$g(t) = \begin{cases} 1 & t \leq 0 \\ a(t) \in (0,1) & 0 < t < \phi^{-1} \\ 0 & t \geq \phi^{-1}, \end{cases}$$

which satisfies that $\frac{da(t)}{dt} \leq \phi$

Step 3 Note $f(\omega) = g \circ F_\beta(\omega) \quad (\omega \in \mathbb{R}^m)$, $V_n := \sqrt{v}S + \sqrt{1-v}G$.

$$\begin{aligned}\sup_{y\in\mathbb{R}^m, v\in[0,1]} P(\sqrt{v}S + \sqrt{1-v}G < y) &:= \sup_{y\in\mathbb{R}^m, v\in[0,1]} P(V_n < y) \\ &= \sup_{y\in\mathbb{R}^m, v\in[0,1]} P(V_n < y - \phi^{-1}) \\ &= \sup_{y\in\mathbb{R}^m, v\in[0,1]} P(V_n - y + \phi^{-1} < 0).\end{aligned} \tag{S14}$$

Because of the property of $F_\beta(\omega)$, we can get

$$F_\beta(\mathbf{V}_n) \leq \max_{1\leq j\leq m}(V_{nj} - y_j) + \phi^{-1}.$$

Therefore, $P(F_\beta(V_n) < 0) \geq P(\max_{1\leq j\leq m}(V_{nj} - y_j) + \phi^{-1} < 0)$, and $(S14) \leq P(F_\beta(V_n) < 0)$. By the definition of the function g, we can get $g(F_\beta(V_n)) \geq I_{\{F_\beta(V_n)<0\}} \geq 0$, we can get $E[g(F_\beta(V_n))] \geq E[I_{\{F_\beta(V_n)<0\}}] = P(F_\beta(V_n) < 0)$. Then we know $\sup_{y\in\mathbb{R}^m, v\in[0,1]} P(V_n < y - \phi^{-1}) \leq E[f(V_n)]$. Furthermore,

$$\begin{aligned}E[f(V_n)] &= E[f(W)] + E[f(V_n) - f(W)] \\ &:= E[f(W)] + E[I_n],\end{aligned} \tag{S15}$$

where $I_n = f(V_n) - f(W)$. Because $g(\omega) \leq I(\omega_j - \phi^{-1} \leq 0)$, we can get $E[f(W)] = E[g(F_\beta(W))] \leq E[I(\max_{1\leq j\leq m}(W_j - y_j) \leq \phi^{-1})]$. So, $E[f(V_n)] \leq P(W \leq y + \phi^{-1}) + E[I_n]$. By **Lemma A.1**, $P(W \leq y + \phi^{-1}) \leq P(W \leq y - \phi^{-1}) + C\phi^{-1}\sqrt{\log m}$. Then, we can get

$$P(V_n < y - \phi^{-1}) \lesssim P(W < y - \phi^{-1}) + C\phi^{-1}\sqrt{\log m} + E[I_n].$$

Similarly, we can get

$$P(V_n < y - \phi^{-1}) \gtrsim P(W < y - \phi^{-1}) - C\phi^{-1}\sqrt{\log m} + E[I_n].$$

In conclusion,

$$\sup_{y\in\mathbb{R}^m, v\in[0,1]} |P(V_n < y - \phi^{-1}) - P(W < y - \phi^{-1})| \lesssim C\phi^{-1}\sqrt{\log m} + |E[I_n]|.$$

Now, Finding the upper bound on ρ_2 translates to finding the upper bound on $E[I_n]$.

Step 4 Let

$$Y_{ij}^* = \frac{n_1+n_2}{n_1}\frac{1}{\sqrt{\sigma_{jj}}}(X_{ij}^1 - \mu_{X:j}^1) \quad (i = 1\cdots n_1; j = 1,\cdots,m),$$

$$Y_{qj}^* = \frac{n_1+n_2}{n_2}\frac{1}{\sqrt{\sigma_{jj}}}[-(X_{ij}^2 - \mu_{X:j}^2)] \quad (q = n_1+1\cdots n_1+n_2; j = 1,\cdots,m).$$

And $Y_i^* = (Y_{i1}^*,\cdots,Y_{im}^*)^T$. Obviously, $Y_1^*,\cdots,Y_{n_1+n_2}^*$ are distributed independently.

$$\begin{aligned}\frac{\bar{X}_j^1 - \bar{X}_j^2 - (\mu_{X:j}^1 - \mu_{X:j}^2)}{\sqrt{\sigma_{jj}}} &= \frac{1}{n_1}\sum_{i=1}^{n_1}\frac{X_{ij}^1 - \mu_{X:j}^1}{\sqrt{\sigma_{jj}}} + \frac{1}{n_2}\sum_{i=1}^{n_2}\frac{-(X_{ij}^2 - \mu_{X:j}^2)}{\sqrt{\sigma_{jj}}} \\ &= \frac{1}{n_1+n_2}\sum_{i=1}^{n_1+n_2} Y_{ij}^* = \bar{X}_j^{2*} \quad (j = 1,\cdots,m).\end{aligned} \tag{S16}$$

Let

$$G_{ij} = \frac{n_1+n_2}{n_1}\frac{1}{\sqrt{\sigma_{jj}}}(G_{X^1:ij} - \mu_{X:j}^1) \quad (i = 1\cdots n_1; j = 1,\cdots,m),$$

$$G_{qj} = \frac{n_1+n_2}{n_2}\frac{1}{\sqrt{\sigma_{jj}}}[-(G_{X^2:ij} - \mu_{X:j}^2)] \quad (q = n_1+1\cdots n_1+n_2; j = 1,\cdots,m).$$

And $G_i = (G_{i1},\cdots,G_{im})^T$. Obviously, $G_1,\cdots,G_{n_1+n_2}$ are distributed independently.

$$\begin{aligned}\frac{\bar{X}_j^1 - \bar{X}_j^2 - (\mu_{X:j}^1 - \mu_{X:j}^2)}{\sqrt{\sigma_{jj}}} &= \frac{1}{n_1}\sum_{i=1}^{n_1}\frac{X_{ij}^1 - \mu_{X:j}^1}{\sqrt{\sigma_{jj}}} + \frac{1}{n_2}\sum_{i=1}^{n_2}\frac{-(X_{ij}^2 - \mu_{X:j}^2)}{\sqrt{\sigma_{jj}}} \\ &= \frac{1}{n_1+n_2}\sum_{i=1}^{n_1+n_2} Y_{ij}^* = \bar{Y}_j^* \quad (j = 1,\cdots,m).\end{aligned} \tag{S17}$$

As a result, L_n and $M_{n,X^1,X^2}(\phi)$ can be written as

$$L_n = \max_{1\le j\le m} \frac{\sum_{i=1}^{n} E[|Y_{ij}^*|^3]}{n},$$

$$M_{n,X^1,X^2}(\phi) = \frac{\sum_{i=1}^{n} E[\max_{1\le j\le m} |Y_{ij}^*|^3 I(|Y_{ij}^*|> \frac{\sqrt{n}}{4\phi\log m})]}{n}.$$

Similarly, define $M_{n,G}(\phi)$ by replacing Y_{ij}^* into G_{ij}.

Define

- $A(t) = \sum_{i=1}^{n} A_i(t), \quad (t \in [0,1])$.
- $A_i(t) = \frac{1}{\sqrt{n}}[\sqrt{t}(\sqrt{v}Y_i^* + \sqrt{1-v}G_i) + \sqrt{1-t}W_i]$.
- $A^{(i)}(t) = A(t) - A_i(t)$ obviously, $A^{(i)}(t)$ and $A_i(t)$ are independent.
- $\dot{A}_i(t) = \frac{1}{\sqrt{n}}[\frac{1}{\sqrt{t}}(\sqrt{v}Y_i^* + \sqrt{1-v}G_i) - \frac{1}{\sqrt{1-t}}W_i]$.

When t=1, we have $A_i(1) = \frac{1}{\sqrt{n}}(\sqrt{v}Y_i^* + \sqrt{1-v}G_i)$, and when t=0, $A_i(0) = \frac{1}{\sqrt{n}}W_i$. Moreover, define the functions

$$h(\omega,t) := I\left\{-\phi^{-1} - \frac{t}{\beta} < \max_{1\le j\le m}(\omega_j - y_j) \le \phi^{-1} + \frac{t}{\beta}\right\}, \omega \in \mathbb{R}^m, t > 0,$$

$$\omega(t) = \frac{1}{\sqrt{t}\wedge\sqrt{1-t}}, t \in (0,1),$$

$$\begin{aligned} I_n &= f(\sqrt{v}S + \sqrt{1-v}G) - f(W) \\ &= f(A(1)) - f(A(0)) \\ &= \int_0^1 \frac{df(A(t))}{dt}dt. \end{aligned} \tag{S18}$$

By Taylor's theorem ,

$$\begin{aligned} E[I_n] &= \frac{1}{2}\sum_{j=1}^{m}\sum_{i=1}^{n}\int_0^1 E[\partial_j f(A(t))\dot{A}_{ij}(t)]dt \\ &= \frac{1}{2}\sum_{j=1}^{m}\sum_{i=1}^{n}\int_0^1 E[\partial_j f(A_i(t)+A^{(i)}(t))\dot{A}_{ij}(t)]dt \\ &= \frac{1}{2}(I+II+III), \end{aligned} \tag{S19}$$

where

- $I = \sum_{i=1}^{n}\sum_{j=1}^{m}\int_0^1 E[\partial_j f(A^{(i)}(t))\dot{A}_{ij}(t)]dt$,
- $II = \sum_{i=1}^{n}\sum_{j=1}^{m}\sum_{k=1}^{m}\int_0^1 E[\partial_j\partial_k f(A^{(i)}(t))A_{ik}(t)\dot{A}_{ij}(t)]dt$,
- $III = \frac{1}{2}\sum_{i=1}^{n}\sum_{j=1}^{m}\sum_{k=1}^{m}\sum_{l=1}^{m}\int_0^1 E[\partial_j\partial_k\partial_l f(A^{(i)}(t)+\theta A_i(t))A_{ik}(t)A_{il}(t)\dot{A}_{ij}(t)]dt \quad (\theta \in (0,1))$.

Next, we bound I,II,III separately,

I

$A_i(t)$ and $A^{(i)}(t)$ are independent , and under $H_0^{(4)}$, we have $E[\dot{A}_{ij}(t)] = 0$, so,

$$E[\partial_j f(A^{(i)}(t))\dot{A}_{ij}(t)] = E[\partial_j f(A^{(i)}(t))] \times E[\dot{A}_{ij}(t)] = 0.$$

II

Because $A_i(t)$ and $A^{(i)}(t)$ are independent and Y_i^*, G_i, W_i are independent, therefore,

$$E[\partial_j\partial_k f(A^{(i)}(t))A_{ik}(t)\dot{A}_{ij}(t)] = E[[\partial_j\partial_k f(A^{(i)}(t))] \times E[A_{ik}(t)\dot{A}_{ij}(t)],$$

and under $H_0^{(4)}$,

$$\begin{aligned} E[A_{ik}(t)\dot{A}_{ij}(t)] &= \frac{1}{n}E[(\sqrt{v}Y_{ij}^* + \sqrt{1-v}G_{ij})(\sqrt{v}Y_{ik}^* + \sqrt{1-v}G_{ik}) - W_{ij}W_{ik}] \\ &= \frac{1}{n}E[vY_{ij}^*Y_{ik}^* + (1-v)G_{ij}G_{ik} - W_{ij}W_{ik}], \end{aligned} \tag{S20}$$

because

$$E[Y_{ij}^*Y_{ik}^*] = E[G_{ij}G_{ik}] = E[W_{ij}W_{ik}],$$

we can get $III = 0$

III

Let $\chi_i = I\{\max_{1\le j\le m} |Y_{ij}^*| \vee |G_{ij}| \vee |W_{ij}| \le \frac{\sqrt{n}}{4\beta}\}$ $(i = 1 \dots n)$, then III can be decomposed into

$$\begin{aligned} III &= \frac{1}{2}\sum_{i=1}^{n}\sum_{j,k,l=1}^{m}\int_0^1 E[\chi_i\partial_j\partial_k\partial_l f(A^{(i)}(t) + \theta A_i(t))A_{ik}(t)A_{il}(t)\dot{A}_{ij}(t)]dt \\ &+ \frac{1}{2}\sum_{i=1}^{n}\sum_{j,k,l=1}^{m}\int_0^1 E[(1-\chi_i)\partial_j\partial_k\partial_l f(A^{(i)}(t) + \theta A_i(t))A_{ik}(t)A_{il}(t)\dot{A}_{ij}(t)]dt \\ &:= III_1 + III_2. \end{aligned} \tag{S21}$$

III_2

$$
\begin{aligned}
| III_2| &\leq \frac{1}{2}\sum_{i=1}^{n}\sum_{j,k,l=1}^{m}\int_0^1 E[(1-\chi_i)U_{jkl}(A^{(i)}(t)+\theta A_i(t))|A_{ik}(t)A_{il}(t)\dot{A}_{ij}(t)|]dt \\
&\textcircled{1} \lesssim \frac{1}{2}\phi\beta^2\sum_{i=1}^{n}\int_0^1 E[(1-\chi_i)\max_{1\leq j,k,l\leq m}|A_{ik}(t)A_{il}(t)\dot{A}_{ij}(t)|]dt \\
&\textcircled{2} \lesssim \frac{\phi\beta^2}{2n^{\frac{3}{2}}}\sum_{i=1}^{n}\int_0^1 w(t)E[(1-\chi_i)\max_{1\leq j\leq m}| Y_{ij}^{*}|^3\vee | G_{ij}|^3\vee | W_{ij}|^3]dt.
\end{aligned}
\tag{S22}
$$

The inequality $\textcircled{1}$ is because **Lemma A.5** :

$$
\begin{gathered}
| \partial_{jkl}f(\omega)| \leq U_{jkl}(\omega), \\
\sum_{j,k,l=1}^{m} U_{jkl}(\omega) \lesssim \phi^3+\phi\beta+\phi\beta^2 \lesssim \phi\beta^2.
\end{gathered}
$$

Note $\Lambda = \dfrac{\sqrt{n}}{4\beta}$.

$$
\begin{aligned}
&\frac{\phi\beta^2}{2n^{\frac{3}{2}}}\sum_{i=1}^{n}\int_0^1 w(t)E[(1-\chi_i)\max_{1\leq j\leq m}| Y_{ij}^{*}|^3\vee | G_{ij}|^3\vee | W_{ij}|^3]dt \\
&\textcircled{3} \lesssim \frac{\phi\beta^2}{2n^{\frac{3}{2}}}\sum_{i=1}^{n}\int_0^1 w(t)dt\times\{E[\max_{1\leq j\leq m}| Y_{ij}^{*}|^3 I\{\max_{1\leq j\leq m}| Y_{ij}^{*}|^3>\Lambda\}]+E[\max_{1\leq j\leq m}| G_{ij}|^3 I\{\max_{1\leq j\leq m}| G_{ij}|^3 \\
&\lesssim \frac{\phi\beta^2}{\sqrt{n}}(M_{n,X^1,X^2}(\phi)+M_{n,G}(\phi)).
\end{aligned}
\tag{S23}
$$

Now, we prove $\textcircled{3}$. Because

$$
\begin{aligned}
1-\chi_i &= I\{\max_{1\leq j\leq m}| Y_{ij}^{*}|\vee | G_{ij}|\vee | W_{ij}|>\Lambda\} \\
&\leq I\{\max_{1\leq j\leq m}| Y_{ij}^{*}|>\Lambda\}+I\{\max_{1\leq j\leq m}| G_{ij}|>\Lambda\}+I\{\max_{1\leq j\leq m}| W_{ij}|>\Lambda\},
\end{aligned}
\tag{S24}
$$

$$\max_{1\le j\le m} \mid Y_{ij}^{*}|^3 \vee \mid G_{ij}|^3 \vee \mid W_{ij}|^3 \le \max_{1\le j\le m} \mid Y_{ij}^{*}|^3 + \max_{1\le j\le m} \mid G_{ij}|^3 + \max_{1\le j\le m} \mid W_{ij}|^3, \tag{S25}$$

$\max_{1\le j\le m} \mid G_{ij}| = \max_{1\le j\le m} \mid W_{ij}|$, By **Lemma A.2**, we can get the inequality ③.

III$_1$

$$\begin{aligned}
III_1 &= \frac{1}{2}\sum_{i=1}^{n}\sum_{j,k,l=1}^{m}\int_0^1 E[\chi_i \partial_j\partial_k\partial_l f(A^{(i)}(t)+\theta A_i(t))A_{ik}(t)A_{il}(t)\dot{A}_{ij}(t)]dt \\
&= \frac{1}{2}\sum_{i=1}^{n}\sum_{j,k,l=1}^{m}\int_0^1 E[\chi_i \mid \partial_j\partial_k\partial_l f(A^{(i)}(t)+\theta A_i(t))A_{ik}(t)A_{il}(t)\dot{A}_{ij}(t)|]dt \\
&\overset{①}{\lesssim} \sum_{i=1}^{n}\sum_{j,k,l=1}^{m}\int_0^1 E[\chi_i h(A^{(i)}(t),1)U_{jkl}(A^{(i)}(t)+\theta A_i(t)) \mid A_{ik}(t)A_{il}(t)\dot{A}_{ij}(t)|]dt \\
&\overset{②}{\lesssim} \sum_{i=1}^{n}\sum_{j,k,l=1}^{m}\int_0^1 E[\chi_i h(A^{(i)}(t),1)U_{jkl}(A^{(i)}(t)) \mid A_{ik}(t)A_{il}(t)\dot{A}_{ij}(t)|]dt \\
&\overset{③}{\lesssim} \sum_{i=1}^{n}\sum_{j,k,l=1}^{m}\int_0^1 E[h(A^{(i)}(t),1)U_{jkl}(A^{(i)}(t))]E[\mid A_{ik}(t)A_{il}(t)\dot{A}_{ij}(t)|]dt.
\end{aligned} \tag{S26}$$

To prove the inequality ①, we should prove when $h(A^{(i)}(t),1)=0$ and $\chi_i=1$, there must have $\partial_{jkl}f(A^{(i)}(t)+\theta A_i(t))=0$ for any $\theta\in[0,1]$. when $\chi_i=1$, namely $\max_{1\le j\le m} \mid Y_{ij}^{*}|\vee \mid G_{ij}|\vee \mid W_{ij}| \le \Lambda$, because $A_{ij}=\frac{1}{\sqrt{n}}[\sqrt{t}(\sqrt{v}Y_{ij}^{*}+\sqrt{1-v}G_{ij})+\sqrt{1-t}W_{ij}]$, we can get $\max_{1\le j\le m} \mid A_{ij}| \le \frac{3}{4\beta} \le \frac{1}{\beta}$. By the definition of $h(\omega,t)$, we can get

$$h(A^{(i)}(t),1) = I\left\{-\phi^{-1}-\frac{1}{\beta} < \max_{1\le j\le m}(A_j^{(i)}(t)-y_j) \le \phi^{-1}+\frac{1}{\beta}\right\},$$

$$h(A^{(i)}(t)+\theta A_i(t),0) = I\left\{-\phi^{-1} < \max_{1\le j\le m}(A_j^{(i)}(t)+\theta A_{ij}(t)-y_j) \le \phi^{-1}\right\}.$$

So, when $h(A^{(i)}(t),1) = 0$, there exists $-\phi^{-1} - \frac{1}{\beta} \geq \max_{1\leq j\leq m}(A_j^{(i)}(t) - y_j)$ or $\max_{1\leq j\leq m}(A_j^{(i)}(t) - y_j) > \phi^{-1} + \frac{1}{\beta}$. Because $\max_{1\leq j\leq m} \mid \theta A_{ij}| < \frac{1}{\beta}$, we can get $-\phi^{-1} \geq \max_{1\leq j\leq m}(A_j^{(i)}(t) + \theta A_{ij} - y_j)$ or $\max_{1\leq j\leq m}(A_j^{(i)}(t) + \theta A_{ij} - y_j) > \phi^{-1}$, namely $h(A^{(i)}(t) + \theta A_i(t), 0) = 0$. Because $\max_{1\leq j\leq m}(\omega_j - y_j) \leq F_\beta(\omega) \leq \phi^{-1} + \max_{1\leq j\leq m}(\omega_j - y_j)$, we can get $F_\beta(A^{(i)}(t) + \theta A_i(t)) \leq 0$ or $F_\beta(A^{(i)}(t) + \theta A_i(t)) \geq \phi^{-1}$. So $f(A^{(i)}(t) + \theta A_i(t))$=0 or 1 for any $\theta \in [0,1]$, we can get $\partial_{jkl} f(A^{(i)}(t) + \theta A_i(t))$=0 for any $\theta \in [0,1]$. Then, we can get ①. ② is true because when $\chi_i = 1, \max_{1\leq j\leq m} \mid A_{ij}| < \frac{1}{\beta}$, we have $U_{jkl}(A^{(i)}(t)) \lesssim U_{jkl}(A^{(i)}(t) + \tau A_i(t)) \lesssim U_{jkl}(A^{(i)}(t))$ from **Lemma A.6**. ③ is true because $\chi_i \leq 1$ and $A^{(i)}(t)$ is independent on $A_{ik}(t)A_{il}(t)\dot{A}_{ij}(t)$. Next, we decompose 1 into $(1-\chi_i) + \chi_i$. Note $III_1 \lesssim III_{11} + III_{12}$.

$$
\begin{aligned}
III_{11} &= \sum_{i=1}^{n}\sum_{j,k,l=1}^{m}\int_0^1 E[(1-\chi_i)h(A^{(i)}(t),1)U_{jkl}(A^{(i)}(t))]E[\mid A_{ik}(t)A_{il}(t)\dot{A}_{ij}(t)|]dt \\
&\overset{①}{\lesssim} \phi\beta^2 \sum_{i=1}^{n}\int_0^1 E[1-\chi_i]E[\max_{1\leq j,k,l\leq m} \mid A_{ik}(t)A_{il}(t)\dot{A}_{ij}(t)|]dt \\
&\overset{②}{\lesssim} \frac{\phi\beta^2}{\sqrt{n}}(M_{n,X^1,X^2}(\phi) + M_{n,G}(\phi)).
\end{aligned}
\tag{S27}
$$

① is true because $h(A^{(i)}(t),1)$=0 or 1 , and from **Lemma A.5**, $\sum_{j,k,l=1}^{m} U_{jkl}(A^{(i)}(t)) \lesssim \phi\beta^2$.
The reasoning for ② is the same as for the upper bound of III_2. Next, let's see III_{12},

$$
\begin{aligned}
III_{12} &= \sum_{i=1}^{n}\sum_{j,k,l=1}^{m}\int_0^1 E[\chi_i h(A^{(i)}(t),1)U_{jkl}(A^{(i)}(t))]E[|\ A_{ik}(t)A_{il}(t)\dot{A}_{ij}(t)|]dt \\
&\overset{①}{\lesssim} \sum_{i=1}^{n}\sum_{j,k,l=1}^{m}\int_0^1 E[h(A(t),2)U_{jkl}(A^{(i)}(t))]E[|\ A_{ik}(t)A_{il}(t)\dot{A}_{ij}(t)|]dt \\
&\lesssim \sum_{j,k,l=1}^{m}\int_0^1 E[h(A(t),2)U_{jkl}(A^{(i)}(t))]\sum_{i=1}^{n}E[|\ A_{ik}(t)A_{il}(t)\dot{A}_{ij}(t)|]dt \\
&\overset{②}{\lesssim} \phi\beta^2\int_0^1 E[h(A(t),2)]\max_{1\le j,k,l\le m}\sum_{i=1}^{n}E[|\ A_{ik}(t)A_{il}(t)\dot{A}_{ij}(t)|]dt.
\end{aligned}
\tag{S28}
$$

To prove ①, we should prove when $h(A(t),2) = 0$ and $\chi_i = 1$, there must be $h(A^{(i)}(t),1) = 0$. By the definition of function $h(\omega,t)$, we can get

$$
h(A(t),2) = I\{-\phi^{-1} - \tfrac{2}{\beta} < \max_{1\le j\le m}(A_j(t) - y_j) \le \phi^{-1} + \tfrac{2}{\beta}\}.
$$

So, when $h(A(t),2) = 0$, we have $-\phi^{-1} - \frac{2}{\beta} \ge \max_{1\le j\le m}(A_j(t) - y_j)$ or $\max_{1\le j\le m}(A_j(t) - y_j) > \phi^{-1} + \frac{2}{\beta}$. As before, when $\chi_i = 1$, we have $\max_{1\le j\le m}(|\ A_{ij}|) < \frac{1}{\beta}$. Therefore, $\max_{1\le j\le m}(A_j(t) - A_{ij}(t) - y_j) \le -\phi^{-1} - \frac{1}{\beta}$, namely $\max_{1\le j\le m}(A_j^{(i)}(t) - y_j) \le -\phi^{-1} - \frac{1}{\beta}$. Or $\max_{1\le j\le m}(A_j(t) - A_{ij}(t) - y_j) > \phi^{-1} + \frac{1}{\beta}$, namely $\max_{1\le j\le m}(A_j^{(i)}(t) - y_j) > \phi^{-1} + \frac{1}{\beta}$, which means $h(A^{(i)}(t),1) = 0$. So, ① is true.

② is true because from **Lemma A.5**, we have

$$
\sum_{j,k,l=1}^{m} U_{jkl}(A^{(i)}(t)) \le \phi\beta^2.
$$

To find the upper bound of III_{12}, next, we find the upper bound of $\max_{1\leq j,k,l\leq m} \sum_{i=1}^{n} E[| A_{ik}(t)A_{il}(t)\dot{A}_{ij}(t)|]$ and $E[h(A,2)]$ separately.

$$|A_{ik}(t)A_{il}(t)\dot{A}_{ij}(t)|\lesssim\frac{w(t)}{(n)^{\frac{3}{2}}}(|Y^*_{ij}|^3+|G_{ij}|^3+|W_{ij}|^3+|Y^*_{ik}|^3+|G_{ik}|^3+|W_{ik}|^3+|Y^*_{il}|^3+|G_{il}|^3+|W_{il}|^3), \quad \text{(S29)}$$

where, because $E[Y^*_{ij}] = E[G_{ij}] = E[W_{ij}] = 0$ and Y^*_i, G_i, W_i are independent. Therefore,

$$\begin{aligned}\max_{1\leq j,k,l\leq m} \sum_{i=1}^{n} E[| A_{ik}(t)A_{il}(t)\dot{A}_{ij}(t)|] \textcircled{1} &\lesssim \frac{w(t)}{(n)^{\frac{3}{2}}} \max_{1\leq j\leq m} \sum_{i=1}^{n}(E[| Y^*_{ij}|^3] + E[| G_{ij}|^3]) \\ \textcircled{2} &\lesssim \frac{w(t)}{\sqrt{n}} L_n.\end{aligned} \quad \text{(S30)}$$

$\textcircled{1}$ is true because $E[| G_{ij}|^3] = E[| W_{ij}|^3]$. $\textcircled{2}$ is true because

$$E[| G_{ij}|^3] \lesssim (E[| G_{ij}|^2])^{\frac{3}{2}} = (E[| Y^*_{ij}|^2])^{\frac{3}{2}} \leq E[| Y^*_{ij}|^3].$$

Now let's bound $h(A,2)$.

$$\begin{aligned}A_j &= \frac{1}{\sqrt{n}} \sum_{i=1}^{n}[[\sqrt{t}(\sqrt{v}Y^*_{ij} + \sqrt{1-v}G_{ij}) + \sqrt{1-t}W_{ij}]] \\ &= \frac{1}{\sqrt{n}} \sum_{i=1}^{n}[\sqrt{tv}Y^*_{ij} + \sqrt{t(1-v)}G_{ij} + \sqrt{1-t}W_{ij}] \\ &\overset{d}{=} \frac{1}{\sqrt{n}} \sum_{i=1}^{n}[\sqrt{tv}Y^*_{ij} + \sqrt{1-tv}G_{ij}] \\ &:= \widetilde{V}_n.\end{aligned} \quad \text{(S31)}$$

$$E[h(A,2)] = P(\widetilde{V}_n \in \bar{I}) - P(\widetilde{V}_n \in \bar{\bar{I}}),$$

where $\bar{I} = \phi^{-1} + \frac{2}{\beta} + y$, $\bar{\bar{I}} = -\phi^{-1} - \frac{2}{\beta} + y$. By the defination of ρ_2, plug $v = tv, y = \bar{I}$ into the inequality, we have

$$P(\widetilde{V}_n \leq \bar{I}) \leq P(G \leq \bar{I}) + \rho_2,$$

plug $y = \bar{\bar{I}}$ into the inequality, we have

$$P(\widetilde{V}_n \leq \bar{\bar{I}}) \geq P(G \leq \bar{\bar{I}}) - \rho_2,$$

G is an m-dimensional centered random vector that obeys a Gaussian distribution. By **Lemma A.1**, we can get

$$P(G \leq \bar{I}) - P(G \leq \bar{\bar{I}}) \lesssim \phi^{-1}\sqrt{\log m}.$$

Therefore, $E[h(A,2)] \lesssim \rho_2 + \phi^{-1}\sqrt{\log m}$. In conclusion,

$$\begin{aligned} | E[I_n]| &\leq I + II + III_{11} + III_{12} + III_2 \\ &\lesssim \frac{\phi^2(\log m)^2}{\sqrt{n}}\{\phi L_n\rho_2 + L_n\sqrt{\log m} + \phi(M_{n,X^1,X^2}(\phi) + M_{n,G}(\phi))\}. \end{aligned} \tag{S32}$$

Then,we can get

$$\rho_2 \lesssim \frac{\phi^2(\log m)^2}{\sqrt{n}}\{\phi L_n\rho_2 + L_n\sqrt{\log m} + \phi(M_{n,X^1,X^2}(\phi) + M_{n,G}(\phi))\} + \frac{\sqrt{\log m}}{\phi}$$

up to a constant K that depends only on b_3.

So, we can get

$$\rho_1 \lesssim \frac{\phi^2(\log m)^2}{\sqrt{n}}\{\phi L_n\rho_1 + L_n\sqrt{\log m} + \phi(M_{n,X^1,X^2}(2\phi) + M_{n,G}(2\phi))\} + \frac{\sqrt{\log m}}{\phi}$$

up to a constant $K^{'}$ that depends only on b_3.

Proof of Theorem 1 Let's set the coefficient K_2 of ϕ_n to be $\dfrac{1}{K' \vee 1} \leq 1$, namely

$$\phi_n = \frac{1}{K' \vee 1}\left(\frac{\overline{L}_n^2 \log^4 m}{n}\right)^{-\frac{1}{6}}.$$

Without loss of generality, Let $\phi_n \geq 2$. Let $\phi = \dfrac{\phi_n}{2}$. Put ϕ into **Lemma A.7**'s conclusion. We can change the coefficient on the right-hand side of the inequality to a constant less than 1 by setting $K_1 = 2(K^{'} \vee 1)$,

$$\rho_1 \leq \frac{\rho_1}{8(K' \vee 1)^2} + \frac{3(K^{'} \vee 1)^2 \bar{L}_n^{\frac{1}{3}} \log^{\frac{7}{6}} m}{n^{\frac{1}{6}}} + \frac{M_{n,X^1,X^2}(\phi_n) + M_{n,G}(\phi_n)}{8(K' \vee 1)^2 \bar{L}_n}.$$

Because $\dfrac{1}{8(K^{'} \vee 1)^2} < 1$, we can get

$$\Delta_1 \leq \rho_1 \leq K_1 \left[\left(\frac{\overline{L}_n^2 \log^7 m}{n}\right)^{\frac{1}{6}} + \frac{M_{n,X^1,X^2}(\phi_n) + M_{n,G}(\phi_n)}{\overline{L}_n}\right],$$

where the constant K_1 depends only on b_3.

Proof of Proposition 1

- Condition D1: $\dfrac{B_n^2 \log^7(mn)}{n} \leq c := \min\{\left(\frac{c_1}{2}\right)^3, \left(\frac{K_2}{2}\right)^6\}$, where $c_1 > 0$ just depends on b_3
- Condition D2: $\dfrac{B_n \log^{\frac{3}{2}} m}{n^{\frac{1}{2}-\frac{1}{q}}} \leq \left(\dfrac{K_2}{2}\right)^{\frac{3}{2}}$

Case for Condition A4 Firstly, let's find the $\overline{L}_n$ which satisfies $\overline{L}_n \geq L_n$.

By Condition A1 and A3, we know

$$\frac{(n_1+n_2)^2}{\sigma_{jj}^{\frac{3}{2}}}\left\{\frac{E[|Y_{ij}^{(1)}-\mu_{\mathbf{X}:j}|^3]}{n_1^2}+\frac{E[|Y_{(i-n_1)j}^{(2)}-\mu_{Y:j}|^3]}{n_2^2}\right\}\leq B_n,$$

namely, $L_n \leq B_n$, so we can note $\overline{L}_n = B_n$. Define $\|Y_{ij}^*\|_{\psi_1} = \inf\left\{t>0: E\left[\exp\left\{\frac{|Y_{ij}^*|}{t}\right\}\right]\leq 2\right\}$. By Condition A4, $E\left[\exp\left\{\frac{|Y_{ij}^*|}{B_n}\right\}\right]\leq 2$, then we can get $\|Y_{ij}^*\|_{\psi_1}\leq B_n$. Because $E[(Y_{ij}^*)^2]=E[G_{ij}^2]$, there exists a constant C_1, such that $\|G_{ij}\|_{\psi_1}\leq C_1B_n$. By Lemma 2.2.2 of Van der Vaart (1996), we know that there exists a constant C_2, such that

$$\|\max_{1\leq j\leq m} Y_{ij}^*\|_{\psi_1}\leq C_2B_n\log m,$$

and

$$\|\max_{1\leq j\leq m} G_{ij}\|_{\psi_1}\leq C_2B_n\log m.$$

By Markov's inequality, we can know that

$$P(\max_{1\leq j\leq m}|Y_{ij}^*|>t)<\frac{E[\max_{1\leq j\leq m}|Y_{ij}^*|]}{t},$$

with $\|\max_{1\leq j\leq m} Y_{ij}^*\|_{\psi_1} = \inf\left\{t>0: E\left[\exp\left\{\frac{\max_{1\leq j\leq m}|Y_{ij}^*|}{t}\right\}\right]\leq 2\right\}\leq C_2B_n\log m$, we know that

$$P(\max_{1\leq j\leq m}|Y_{ij}^*|>t)\leq 2\exp\left\{-\frac{t}{C_2B_n\log m}\right\}.$$

By **Lemma A.3**, we can get

$$E\left[\max_{1\leq j\leq m}|Y_{ij}^*|^3 I\left\{\max_{1\leq j\leq m}|Y_{ij}^*|>\frac{\sqrt{n}}{4\phi\log m}\right\}\right]\leq 12\left(\frac{\sqrt{n}}{4\phi\log m}+C_2B_n\log m\right)^3\exp\left\{-\frac{\frac{\sqrt{n}}{4\phi\log m}}{C_2B_n\log m}\right\},$$

namely,

$$M_{n,X^1,X^2}(\phi_n) \lesssim \left(\frac{\sqrt{n}}{\phi_n \log m} + B_n \log m\right)^3 \exp\left\{-\frac{\sqrt{n}}{4C_2\phi_n B_n log^2 m}\right\}.$$

Let $c_1 = \dfrac{1}{4K_2C_2}$, then we can get

$$\frac{\sqrt{n}}{4C_2\phi_n B_n log^2 m} = \frac{c_1 n^{\frac{1}{3}}}{B_n^{\frac{2}{3}} \log^{\frac{4}{3}} m}.$$

By Condition D1, we can get that $\dfrac{c_1 n^{\frac{1}{3}}}{B_n^{\frac{2}{3}} \log^{\frac{4}{3}} m} \geq c_1 c^{-\frac{1}{3}} \log(mn)$. Because $(\dfrac{c_1}{2})^3 > c$, we can get $c_1 c^{-\frac{1}{3}} > 2$, then $\dfrac{c_1 n^{\frac{1}{3}}}{B_n^{\frac{2}{3}} \log^{\frac{4}{3}} m} \geq 2\log(mn)$. By Condition D1, we can get $\phi_n^{-1} \leq K_2^{-1}(\dfrac{B_n^2 \log^4 m}{n})^{\frac{1}{6}} \leq \dfrac{c^{\frac{1}{6}}}{K_2} \leq 1$. Because of the fundemental inequality and Condition D1, we can get

$$\left(\frac{\sqrt{n}}{\phi_n \log m} + B_n \log m\right)^3 \lesssim n^{\frac{3}{2}}.$$

Therefore, $M_{n,X^1,X^2}(\phi_n) \lesssim n^{\frac{3}{2}} \exp\{-2\log(mn)\} = n^{\frac{3}{2}}(\exp\{\log^{-1}(mn)\})^2 \leq \frac{1}{\sqrt{n}}$. The same reasoning also gives $M_{n,G}(\phi_n) \lesssim \frac{1}{\sqrt{n}}$. Therefore, with **Theorem 1**, we can get

$$\begin{aligned}
\Delta_1 &\leq K_1 \left[(\frac{B_n^2 \log^7 m}{n})^{\frac{1}{6}} + \frac{M_{n,X^1,X^2}(\phi_n) + M_{n,G}(\phi_n)}{B_n}\right] \\
&\leq K_1 \left[(\frac{B_n^2 \log^7 m}{n})^{\frac{1}{6}} + \frac{1}{\sqrt{n}} B_n^{-1}\right] \\
&\leq C_1 \left(\frac{B_n^2 \log^7 mn}{n}\right)^{\frac{1}{6}},
\end{aligned} \tag{S33}$$

where the constant C_1 only depends on b_3.

Case for Condition A5 By condition D2, $L_n \leq B_n + \frac{B_n^2}{n^{\frac{1}{2}-\frac{2}{q}}\sqrt{\log m}} := \overline{L}_n$. Therefore, we have

$$
\begin{aligned}
(\frac{\overline{L}_n^2 \log^7 m}{n})^{\frac{1}{6}} &\leq (\frac{B_n^2 \log^7 m}{n} + \frac{B_n^4 \log^6 m}{n^{2-\frac{4}{q}}})^{\frac{1}{6}} \\
&\leq (\frac{B_n^2 \log^7 (mn)}{n})^{\frac{1}{6}} + (\frac{B_n^2 \log^3 (mn)}{n^{1-\frac{2}{q}}})^{\frac{1}{3}} \\
&\leq K_2.
\end{aligned} \tag{S34}
$$

The last inequality is because of Condition D1 and D2. Therefore,

$$
\begin{aligned}
\phi_n^{-1} &= K_2^{-1} (\frac{B_n^2 \log^4 m}{n})^{\frac{1}{6}} \\
&\leq K_2^{-1} (\frac{B_n^2 \log^7 m}{n})^{\frac{1}{6}} \\
&\leq 1.
\end{aligned} \tag{S35}
$$

Note that for any real-valued random variable Z and any $t > 0$,

$$
\begin{aligned}
E[|Z|^3 I_{\{|Z|>t\}}] &\leq E\left[|Z|^3 \left(\frac{|Z|}{t}\right)^{q-3} I_{\{|Z|>t\}}\right] \\
&\leq \left(\frac{1}{t}\right)^{q-3} E[|Z|^q].
\end{aligned} \tag{S36}
$$

Hence

$$
\begin{aligned}
M_{n,X^1,X^2}(\phi) &= \frac{\sum_{i=1}^n E\left[\max_{1\leq j\leq m} |Y_{ij}^*|^3 I\left\{\max_{1\leq j\leq m} |Y_{ij}^*| > \frac{\sqrt{n}}{4\phi \log m}\right\}\right]}{n} \\
&\leq \left(\frac{\sqrt{n}}{4\phi \log m}\right)^{3-q} B_n^q E\left[\max_{1\leq j\leq m} \frac{|Y_{ij}^*|}{B_n}^q\right]
\end{aligned} \tag{S37}
$$

By Condition A5, $E\left[\max_{1\le j\le m}\frac{|Y_{ij}^*|^q}{B_n}\right]\le 2$, so, $M_{n,X^1,X^2}(\phi_n)\lesssim\frac{B_n^q\phi_n^{q-3}\log^{q-3}m}{n^{\frac{q}{2}-\frac{3}{2}}}$.

Because $\overline{L}_n\ge\frac{B_n^2}{n^{\frac{1}{2}-\frac{2}{q}}\sqrt{\log m}}$, we can get $\overline{L}_n^{-1}\le B_n^{-2}n^{\frac{1}{2}-\frac{2}{q}}\sqrt{\log m}$, put it into $\phi_n=K_2\left(\frac{\overline{L}_n^2\log^4 m}{n}\right)^{-\frac{1}{6}}$, we can get $\phi_n\lesssim n^{\frac{1}{3}-\frac{2}{3q}}B_n^{\frac{2}{3}}\frac{1}{\sqrt{\log m}}$. So, $M_{n,X^1,X^2}(\phi_n)\lesssim\frac{B_n^{\frac{q}{3}+2}\log^{\frac{q}{2}-\frac{3}{2}}m}{n^{\frac{q}{6}+\frac{1}{6}-\frac{2}{q}}}$. So, $M_{n,X^1,X^2}(\phi_n)\overline{L}_n^{-1}\le n^{\frac{1}{3}-\frac{q}{6}}B_n^{\frac{q}{3}}\log^{\frac{q}{2}-1}m\lesssim\left(\frac{B_n^2\log^3(mn)}{n^{1-\frac{q}{2}}}\right)^{\frac{1}{3}}$.(q¿ 0 makes the last inequality work) By $M_{n,G}(\phi_n)\lesssim\frac{1}{\sqrt{n}}$, we can get $\frac{M_{n,G}(\phi_n)}{\overline{L}_n}\le\frac{M_{n,G}(\phi_n)}{B_n}\lesssim\left(\frac{B_n^2\log^7(mn)}{n}\right)^{\frac{1}{6}}$. In conclusion,

$$\Delta_1\le C_2\left[\left(\frac{B_n^2\log^7(mn)}{n}\right)^{\frac{1}{6}}+\left(\frac{B_n^2\log^3(mn)}{n^{1-\frac{2}{q}}}\right)^{\frac{1}{3}}\right],$$

where the constant C_2 only depends on q and b_3.

Proof of Corollary 1 By some algebra, we can obtain that

$\sup_{t\in\mathbb{R}}|P(\max_{1\le j\le m}S_j^2<t)-P(\max_{1\le j\le m}G_j^2<t)|$

$=\sup_{t\in\mathbb{R}}|P(S_1^2<t,\dots,S_m^2<t)-P(G_1^2<t,\dots,G_m^2<t)|$

$=\sup_{t\in\mathbb{R}}|P(-\sqrt{t}<S_1<\sqrt{t},\dots,-\sqrt{t}<S_m<\sqrt{t})-P(-\sqrt{t}<G_1<\sqrt{t},\dots,-\sqrt{t}<G_m<\sqrt{t})|$.

$\le\sup_{A\in\mathcal{A}^{re}}|P(S\in A)-P(G\in A)|$

$\le C_1\left(\frac{B_n^2\log^7 mn}{n}\right)^{\frac{1}{6}}$, where the constant C_1 only depends on b_3.

APPENDIX B

Appendix B presents some lemmas and the proof of **Lemma 1** in Supp Section 3.

Lemma B.1 Let real positive sequences $b_m(m = 1, 2, \cdots)$ are satisfied with the following equations:

$$b_m e^{b_m} = \frac{m^2}{2\pi}(m = 1, 2, \cdots).$$

As $m \to \infty$, we have the following asymptotic expansion:

$$b_m = 2\log m - [\log(\log m) + \log(4\pi)] + \frac{\log(\log m) + \log(4\pi)}{2\log m} + o(\frac{1}{\log m}).$$

Proof Obviously, b_m is a positive monotone incremental sequence with $\lim_{m\to\infty} b_m = \infty$. According to the equation in the Lemma B.1, we have

$$\log b_m + b_m = 2\log m - \log(2\pi),$$

the both above sides divided by b_m, we have

$$\frac{\log b_m}{b_m} + 1 = \frac{2\log m}{b_m} - \frac{\log(2\pi)}{b_m}.$$

Because $\lim_{m\to\infty} \frac{\log b_m}{b_m} = 0$, so

$$\lim_{m\to\infty} \frac{2\log m}{b_m} = 1.$$

Denote $a_m = \frac{b_m}{2\log m} - 1$, we have $\lim_{m\to\infty} a_m = 0$, and $b_m = 2(1 + a_m)\log m$. Further more,

$$\log(1 + a_m) + \log(2\log m) + b_m = 2\log m - \log(2\pi).$$

According to $b_m = 2\log m - [\log(\log m) + \log(4\pi)] - \log(1 + a_m)$, and $b_m = 2(1 + a_m)\log m$, we can have

$$a_m = -\frac{\log(\log m)}{2\log m} - \frac{\log(4\pi)}{2\log m} - \frac{\log(1 + a_m)}{2\log m},$$

and

$$a_m^2 = o(\frac{1}{\log m}).$$

According to the Tylor expansion of $\log(1 + a_m)$, we can have

$$b_m = 2\log m - [\log(\log m) + \log(4\pi)] - a_m + \frac{a_m^2}{2} + o(a_m^2).$$

Eventually, we have

$$b_m = 2\log m - [\log(\log m) + \log(4\pi)] + \frac{\log(\log m) + \log(4\pi)}{2\log m} + o(\frac{1}{\log m}).$$

This completes the proof **Lemma B.1**.

Lemma B.2 Let $Z_1, \cdots, Z_m$ independently follow a common standard normal distribution. We can have, $\forall x \in R$,

$$\lim_{m\to\infty} P\left(\frac{[\max\limits_{s=1,\cdots,m}(|Z_s|)]^2 - c_m}{2 - (\log m)^{-1}} < x\right) = \exp\left(-e^{-x}\right),$$

where $c_m = 2\log m - [\log\left(\log m\right) + log(4\pi) - \log 4] + \frac{\log\left(\log m\right) + log(4\pi)\log 4}{2\log m}$.

Proof Denote $M_m = \max\limits_{s=1,\cdots,m}(|Z_s|)$ and $P\left(M_m < y\right) = P\left(\max\limits_{s=1,\cdots,m}(|Z_s|) < y\right) = [F(y)]^m$, where $F(y) = [2\Phi(y) - 1]I(y > 0)$ is the cumulative distribution function of $|Z_s|$, and $\Phi(\cdot)$ is the cumulative distribution probability function of a standard normal random variable. Denote $W_m = m\{1 - F[\max\limits_{s=1,\cdots,m}(|Z_s|)]\}$ and $H(w)$ the probability function of

W_m. For any $w > 0$, we have

$$H(w) = P(W_m < w) = P\left(m\{1 - F[\max_{1,\cdots,m}(|Z_s|)]\} < w\right) = 1 - \left(1 - \frac{w}{m}\right)^m.$$

Denote $h(w)$ the density function of W_m. For any $w > 0$, we have

$$h(w) = \left(1 - \frac{w}{m}\right)^{m-1}.$$

For any real number $w > 0$, we can have

$$\lim_{m\to\infty} h(w) = e^{-w}.$$

According to the definition of W_m, we can have

$$W_m = \frac{m}{\sqrt{2\pi}} \int_{M_m}^{\infty} (2e^{-\frac{t^2}{2}})dt.$$

By partial integration, the equation may be put in the form

$$\frac{W_m\sqrt{2\pi}}{m} = \frac{2}{M_m} e^{-\frac{M_m^2}{2}} [1 + O(\frac{1}{M_m^3})],$$

namely,

$$M_m^2 e^{M_m^2} = \frac{m^2}{2\pi \frac{W_m^2}{4}} [1 + O(\frac{1}{M_m^3})]^2.$$

According to **Lemma B.1**, we can have $M_m^2 = 2\log m - [\log(\log m) + \log(4\pi) - \log 4 + 2\log W_m] + \frac{\log(\log m)+\log(4\pi)-\log 4+2\log W_m}{2\log m} + o(\frac{1}{\log m})$, namely, $\frac{M_m^2-\{2\log m-[\log(\log m)+\log(4\pi)-\log 4]+\frac{\log(\log m)+\log(4\pi)-\log 4m}{2\log m}\}}{2-(\log m)^{-1}} = -\log W_m + o(\frac{1}{\log m})$. So, $\forall x \in R$, $\lim_{m\to\infty} P\left(\frac{M_m^2-\{2\log m-[\log(\log m)+log(4\pi)-\log 4]+\frac{\log(\log m)+log(4\pi)-\log 4}{2\log m}\}}{2-(\log m)^{-1}} < x\right) = \lim_{m\to\infty} P\left(-\log W_m < x\right)$

$= \lim_{m\to\infty} P\left(W_m \geq e^{-x}\right) = \exp\left(-e^{-x}\right)$, This completes the proof **Lemma B.2**.

Proof of Lemma 1 Let x a positive real number, and

$$d_m = [2-(\log m)^{-1}]x+\left\{2\log m - [\log(\log m) + log(4\pi) - \log 4] + \frac{\log(\log m) + log(4\pi) - \log 4}{2\log m}\right\}.$$

Consider a auxiliary Gaussian sequence $\{Y_m : 1 \leq m < \infty\}$ of random variables which are independent have means zero and variance one. We shall show that under the conditions of the Lemma,

$$\left|P\left([\max_{s=1,\cdots,m}|G_s|]^2 < d_m\right) - P\left([\max_{s=1,\cdots,m}(|Y_s|)]^2 < d_m\right)\right| \to 0, \ \ as \ \ m \to \infty.$$

This, together with **Lemma B.2**, will complete the proof of this **Lemma 1**. Now

$$\left|P\left([\max_{s=1,\cdots,m}|G_s|]^2 < d_m\right) - P\left([\max_{s=1,\cdots,m}(|Y_s|)]^2 < d_m\right)\right|$$

$$= \left|P\left(\max_{s=1,\cdots,m}|G_s| < \sqrt{d_m}\right) - P\left(\max_{s=1,\cdots,m}(|Y_s|) < \sqrt{d_m}\right)\right|$$

$$= \left|P\Big(-\sqrt{d_m} < G_s < \sqrt{d_m}\,; 1 \leq s \leq m\Big) - P\Big(-\sqrt{d_m} < Y_s < \sqrt{d_m}\,; 1 \leq s \leq m\Big)\right|.$$

According to **Lemma B.2**, Lemma 11.1.2(Page 207) in Leadbetter et al.(1983) and condition B1, we complete the proof **Lemma 1**.

APPENDIX C

Appendix C presents the proofs of **Proposition 2**, **Proposition 3**, **Proposition 4**, **Lemma 3**, **Lemma 4**, **Theorem 2** and **Theorem 3** in Supp. section 4.

Lemma C.1 Under Condition C4, there exist constants $c_1, c_2, c_3, c_4 > 0$, such that

$$P\left(\max_{k,j}\frac{|\hat{\sigma}_{L^g:kj}-\sigma^g_{L:kj}|}{\sqrt{(\sigma^g_{L:kk}\sigma^g_{L:jj})}}\geq t\right)\leq c_1\exp\left\{-\frac{c_2 n_g t}{2}\right\}+c_3 m^2\exp\left\{-\frac{c_4 n_g t^2}{4}\right\}\quad(t>0)(g=1,2)$$

Proof Please refer to Bickel and Levina (2008).

Proof of Proposition 2 For a matrix $A=(a_{ij})_{m\times m}$, write $\parallel A \parallel_1 = \max\limits_{1\leq j\leq m}\sum\limits_{i=1}^{m}|a_{ij}|$, $a_{i\cdot}=\frac{1}{m}\sum\limits_{j=1}^{m}a_{ij}$, $a_{\cdot j}=\frac{1}{m}\sum\limits_{i=1}^{m}a_{ij}$, $a_{\cdot\cdot}=\frac{1}{m^2}\sum\limits_{i=1}^{m}\sum\limits_{j=1}^{m}a_{ij}$. By Condition C3, we can get

$$\begin{aligned}\|R_{L^1}\|_1 &= \max_{1\leq j\leq m}\sum_{i=1}^{m}|\rho_{L^1:ij}|\\ &\textcircled{1}\leq \sqrt{m}\max_{1\leq j\leq m}\sqrt{\sum_{i=1}^{m}(\rho_{L^1:ij})^2} \qquad \text{(S38)}\\ &\leq \sqrt{mb_6}\\ &= O(\sqrt{m}).\end{aligned}$$

Similarly, we can also get $\|R_{L^2}\|_1 \leq O(\sqrt{m})$. The inequality ① is true because By Condion C2, $|\rho_{L^1:ij}|, |\rho_{L^2:ij}| < 1$ for any $i, j \in (1, \ldots, m)$. By Condition C1, we know

$$\begin{aligned}
|\sigma_{L^1:i\cdot}| &= |\frac{1}{m}\sum_{j=1}^{m}\sigma_{L^1:ij}| \\
&\leq \frac{1}{m}\sum_{j=1}^{m}|\sigma_{L^1:ij}| \\
② &\leq \frac{1}{m}\max_{1\leq j\leq m}|\sigma^1_{L:jj}|\sum_{j=1}^{m}|\rho_{L^1:ij}| \\
&\leq \frac{1}{m}\kappa_1\|R_{X^1}\|_1 \\
&\leq O\left(\frac{1}{\sqrt{m}}\right).
\end{aligned} \tag{S39}$$

Similarly, we can also get $|\sigma_{L^2:i\cdot}| \leq O\left(\frac{1}{\sqrt{m}}\right)$. The inequality ② is true because $|\rho_{L^1:ij}| = \left|\frac{\sigma_{L^1:ij}}{\sqrt{\sigma_{L^1:ii}}\sqrt{\sigma^1_{L:jj}}}\right| \geq \max_{1\leq j\leq m}\frac{|\sigma_{L^1:ij}|}{|\sigma^1_{L:jj}|}$ and $|\rho_{L^2:ij}| = |\frac{\sigma_{L^2:ij}}{\sqrt{\sigma_{L^2:ii}}\sqrt{\sigma^2_{L:jj}}}| \geq \max_{1\leq j\leq m}\frac{|\sigma_{L^2:ij}|}{|\sigma^2_{L:jj}|}$ for any i $\in (1, \ldots, m)$.

Similarly, we can get $|\sigma_{L^1:\cdot j}| := |\frac{1}{m}\sum_{i=1}^{m}\sigma_{L^1:ij}| = O\left(\frac{1}{\sqrt{m}}\right)$,$|\sigma_{L^1:\cdot\cdot}| := |\frac{1}{m^2}\sum_{i=1}^{m}\sum_{j=1}^{m}\sigma_{L^1:ij}| = O\left(\frac{1}{\sqrt{m}}\right)$ and $|\sigma_{L^2:\cdot j}| := |\frac{1}{m}\sum_{i=1}^{m}\sigma_{L^2:ij}| = O\left(\frac{1}{\sqrt{m}}\right)$,$|\sigma_{L^2:\cdot\cdot}| := |\frac{1}{m^2}\sum_{i=1}^{m}\sum_{j=1}^{m}\sigma_{L^2:ij}| = O\left(\frac{1}{\sqrt{m}}\right)$.

Because of the equality $\Sigma^1_X = U\Sigma^1_L U^T$ and $\Sigma_Y = U\Sigma^2_L U^T$, we can get

$$\sigma_{X^1:ij} = \sigma_{L^1:ij} - \sigma_{L^1:i\cdot} - \sigma_{L^1:\cdot j} + \sigma_{L^1:\cdot\cdot},$$

$$\sigma_{X^2:ij} = \sigma_{L^2:ij} - \sigma_{L^2:i\cdot} - \sigma_{L^2:\cdot j} + \sigma_{L^2:\cdot\cdot}.$$

Therefore,

$$\begin{aligned}\|\Sigma_X^1 - \Sigma_L^1\|_{\max} &\le \max_{1\le i,j\le m} (|\sigma_{L^1:i\cdot}| + |\sigma_{L^1:\cdot j}| + |\sigma_{L^1:\cdot\cdot}|) \\ &= O\left(\frac{1}{\sqrt{m}}\right),\end{aligned} \tag{S40}$$

and we can also get $\|\Sigma_X^2 - \Sigma_L^2\|_{\max} \le O\left(\frac{1}{\sqrt{m}}\right)$ which, together with Condition C1, implies Proposition 2.

Proof of Proposition 3 Under $H_0^{(4)}$, without loss of generality, we assume that $\mu_{X^1} = \mu_{X^2} = 0$, because $\Sigma_{X^g} = U\Sigma_{L^g}U^T$ (g=1,2) , we can get

$$|\ \hat{\sigma}_{X^g:jj} - \sigma^g_{X:jj}| = |\hat{\sigma}_{L^g:jj} - 2\hat{\sigma}_{L^g:j\cdot} + \hat{\sigma}_{L^g:\cdot\cdot} - (\sigma^g_{L:jj} - 2\sigma_{L^g:j\cdot} + \sigma_{L^g:\cdot\cdot})| \le 4\max_{i,j} |\hat{\sigma}_{L^g:ij} - \sigma_{L^g:ij}| (g = 1, 2).$$

Therefore,

$$\frac{|\ \hat{\sigma}_{X^g:jj} - \sigma^g_{X:jj}|}{\sigma^g_{X:jj}} \le \frac{4}{\sigma^g_{X:jj}} \max_{i,j} \frac{|\hat{\sigma}_{L^g:ij} - \sigma_{L^g:ij}|}{\sqrt{\sigma_{L^g:ii}\sigma^g_{L:jj}}} \sqrt{\sigma_{L^g:ii}\sigma^g_{L:jj}} (g = 1, 2).$$

By Condition C1 and **Proposition 2**, we can get

$$\frac{4}{\sigma^g_{X:jj}} \max_{i,j} \frac{|\hat{\sigma}_{L^g:ij} - \sigma_{L^g:ij}|}{\sqrt{\sigma_{L^g:ii}\sigma^g_{L:jj}}} \sqrt{\sigma_{L^g:ii}\sigma^g_{L:jj}} \le 4\kappa_1\kappa_2 \max_{i,j} \frac{|\hat{\sigma}_{L^g:ij} - \sigma_{L^g:ij}|}{\sqrt{\sigma_{L^g:ii}\sigma^g_{L:jj}}} (g = 1, 2),$$

which, together with **Lemma C.1**, implies

$$\frac{|\ \hat{\sigma}^1_{X:jj} - \sigma^1_{X:jj}|}{\sigma_{X^1:jj}} = O_p\left\{\sqrt{\frac{\log m}{n}}\right\}.$$

$$
\begin{aligned}
\frac{|\sigma_{jj}-\hat{\sigma}_{jj}|}{\sigma_{jj}} &\leq \frac{\dfrac{n_1+n_2}{n_1}|\sigma^1_{X:jj}-\hat{\sigma}^1_{X:jj}|+\dfrac{n_1+n_2}{n_2}|\sigma^2_{X:jj}-\hat{\sigma}^2_{X:jj}|}{\dfrac{n_1+n_2}{n_1}\sigma^1_{X:jj}+\dfrac{n_1+n_2}{n_2}\sigma^2_{X:jj}} \\
&\leq \frac{|\sigma^1_{X:jj}-\hat{\sigma}^1_{X:jj}|}{\sigma^1_{X:jj}}+\frac{|\sigma^2_{X:jj}-\hat{\sigma}^2_{X:jj}|}{\sigma^2_{X:jj}} \qquad \text{(S41)} \\
&= O_p\left\{\sqrt{\frac{\log m}{n}}\right\}.
\end{aligned}
$$

Then we finish the proof.

Proof of Proposition 4 We write

$$
r_{kj}=\frac{\sigma_{kj}}{\sqrt{\sigma_{kk}\sigma_{jj}}}=\frac{\sigma_{L:kj}+\alpha_1}{\sqrt{(\sigma_{L:kk}+\alpha_2)(\sigma_{L:jj}+\alpha_3)}},
$$

where $\alpha_1=-\sigma_{L:k\cdot}-\sigma_{L:\cdot j}+\sigma_{L:\cdot\cdot}$, $\alpha_2=-2\sigma_{L:k\cdot}+\sigma_{L:\cdot\cdot}$ and $\alpha_3=-2\sigma_{L:\cdot j}+\sigma_{L:\cdot\cdot}$. In **Proposition 2**, we know that $|\sigma_{L:k\cdot}|=O\left(\frac{1}{\sqrt{m}}\right)$, $|\sigma_{L:\cdot j}|=O\left(\frac{1}{\sqrt{m}}\right)$, $|\sigma_{L:\cdot\cdot}|=O\left(\frac{1}{\sqrt{m}}\right)$. So, we can get that $\alpha_i=O\left(\frac{1}{\sqrt{m}}\right)$ (i=1,2,3). With Condition B1, we can obtain

$$
r_{kj}=\frac{\sigma_{L:kj}+\alpha_1}{\sqrt{\sigma_{L:kk}\sigma_{L:jj}}}\left\{\frac{(\sigma_{L:kk}+\alpha_2)(\sigma_{L:jj}+\alpha_3)}{\sigma_{L:kk}\sigma_{L:jj}}\right\}^{-\frac{1}{2}}=\frac{r_{L:kj}+O\left(\frac{1}{\sqrt{m}}\right)}{1+O\left(\frac{1}{\sqrt{m}}\right)}=r_{L:ij}+O\left(\frac{1}{\sqrt{m}}\right),
$$

which, with Condition C6, implies **Proposition 4**.

Proof of Lemma 2 Denote $d_m(t):=(2-(\log m)^{-1})t+c_m$

Obviously, $[\max_{s=1,\cdots,m}(|G_j|)]^2=\max_{s=1,\cdots,m}(G_j^2)$. According to **Lemma 1** ,we can have

$$
\lim_{n_1,n_2,m\to\infty}P\left(\frac{1}{2-(\log m)^{-1}}[\max_{1\leq j\leq m}G_j^2-(c_m+\log 4-\frac{\log 4}{2\log m})]<t\right)=\exp(-\exp(-t)).
$$

Namely, $\lim_{n_1,n_2,m\to\infty}P\Big(\max_{1\leq j\leq m}G_j^2<d_m(t)+2(\log 4-\frac{\log 4}{2\log m})\Big)=\exp(-\exp(-t))$.

According to **Corollary 2**, we can have

$$\lim_{n_1,n_2,m\to\infty} P\left(\max_{1\le j\le m}(S_j)^2 < d_m(t) + 2\left(\log 4 - \frac{\log 4}{2\log m}\right)\right) = \exp(-\exp(-t)).$$

Namely,

$$\lim_{n_1,n_2,m\to\infty} P\left(\frac{\tilde{T}_1 - \left(c_m + \log 4 - \frac{\log 4}{2\log m}\right)}{2-(\log m)^{-1}} < t\right) = \exp(-\exp(-t)).$$

Proof of Theorem 2

$$\begin{aligned} |T_{MECAF} - \tilde{T}_1| &= n\left[\left|\max_{1\le j\le m}\frac{\sigma_{jj}(\bar{X}_j^1 - \bar{X}_j^2)^2}{\hat{\sigma}_{jj}\sigma_{jj}} - \max_{1\le j\le m}\frac{\hat{\sigma}_{jj}(\bar{X}_j^1 - \bar{X}_j^2)^2}{\hat{\sigma}_{jj}\sigma_{jj}}\right|\right] \\ &\le n\max_{1\le j\le m}\frac{(\bar{X}_j^1 - \bar{X}_j^2)^2}{\hat{\sigma}_{jj}}\max_{1\le j\le m}\frac{|\,\hat{\sigma}_{jj} - \sigma_{jj}|}{\sigma_{jj}}. \end{aligned} \tag{S42}$$

By **Proposition 3**, we know that for some constants $C_4 > 0$, we have

$$P\left(\max_{1\le j\le m}\frac{|\,\hat{\sigma}_{jj} - \sigma_{jj}|}{\sigma_{jj}} \le C_4\sqrt{\frac{\log m}{n}}\right) \to 1.$$

With Condition C5, $|T_1 - \tilde{T}_1| \le C_4 T_1\sqrt{\frac{\log m}{n}} = T_1 o\left(\frac{1}{\log m}\right)$. This together with **Lemma 1**, completes the proof.

APPENDIX D

Appendix D presents detailed proof of **Theorem 3** in Supp. Section 5.

Proof of Theorem 3 Denote $\widetilde{\Phi}_{1:\alpha} = I(\widetilde{T}_{MECAF} \geq [2-(\log m)^{-1}]q_{1:\alpha} + c_m)$, the null hypothesis $H_0^{(4)}$ is rejected whenever $\widetilde{\Phi}_{1:\alpha} = 1$. In view of $|T_{MECAF} - \tilde{T}_1| \leq CT_{MECAF}o(\frac{1}{\log m})$, it suffices to prove that under $H_1^{(5)}$,

$$P(\widetilde{T}_1 \geq [2-(\log m)^{-1}]q_{1:\alpha} + c_m) \to 1. \tag{S43}$$

By assumption, there exists some $j_0 \in \xi$ such that $|\delta_{j_0}| \geq \sqrt{2}+\varepsilon$, we write

$$\sqrt{n}\frac{\bar{X}_{j_0}^1 - \bar{X}_{j_0}^2}{\sqrt{\sigma_{j_0j_0}}} = \sqrt{n}\frac{\bar{X}_{j_0}^1 - \mu_{X:j_0}^1}{\sqrt{\sigma_{j_0j_0}}} - \sqrt{n}\frac{-\mu_{X:j_0}^2 + \bar{X}_{j_0}^2}{\sqrt{\sigma_{j_0j_0}}} + \sqrt{n}\frac{\mu_{X:j_0}^1 - \mu_{X:j_0}^2}{\sqrt{\sigma_{j_0j_0}}} := u_1 + u_2 + u_3. \tag{S44}$$

Note that $u_1 = O_p(1)$ and $u_2 = O_p(1)$ by the central limit theorem. Define $u_4 = \sqrt{n}\frac{\mu_{X:j_0}^1 - \mu_{X:j_0}^2}{\sqrt{\sigma_{L:j_0j_0}}}$. It follows from condition B1 and the equation $\| \Sigma - \Sigma_L \|_{\max} \leq \lim_{1\leq i,j\leq m}(|\sigma_{L:i\cdot}| + |\sigma_{L:j\cdot}| + |\sigma_{L:\cdot\cdot}|) = O\left(\frac{1}{\sqrt{m}}\right)$ that

$$|u_3 - u_4| = \sqrt{n}\frac{|\mu_{X:j_0}^1 - \mu_{X:j_0}^2|}{\sqrt{\sigma_{j_0j_0}}}\frac{|\sqrt{\sigma_{j_0j_0}} - \sqrt{\sigma_{L:j_0j_0}}|}{\sqrt{\sigma_{L:j_0j_0}}} = |u_3|O\left(\frac{1}{\sqrt{m}}\right). \tag{S45}$$

Then using the assumption $\| \delta \|_1 = o(m)$, we have

$$\begin{aligned} u_3 &= u_4\left(1 + O\left(\frac{1}{\sqrt{m}}\right)\right) \\ &= \sqrt{n}\frac{\delta_{j_0}\sqrt{\sigma_{L:j_0j_0}} + o(1)}{\sqrt{\sigma_{L:j_0j_0}}}\sqrt{\frac{\log m}{n}}[1 + O(\frac{1}{\sqrt{m}})] \\ &= [\delta_{j_0} + o(1)]\sqrt{\log m} \\ &\geq (\sqrt{2} + \frac{\varepsilon}{2})\sqrt{\log m}, \end{aligned} \tag{S46}$$

for sufficiently large m. Combining these bounds, we conclude that, with probability tending to 1,

$$\sqrt{n}\frac{|\bar{X}_{j_0}^1 - \bar{X}_{j_0}^2|}{\sqrt{\sigma_{j_0 j_0}}} \geq \sqrt{\left[2 - (\log m)^{-1}\right] q_{1:\alpha} + c_m}. \tag{S47}$$

So, we can get

$$P\left(\max_{1\leq j\leq m}\left(\sqrt{n}\frac{\bar{X}_{j_0}^1 - \bar{X}_{j_0}^2}{\sqrt{\sigma_{j_0 j_0}}}\right)^2 \geq \left[2 - (\log m)^{-1}\right] q_{1:\alpha} + c_m\right) \to 1. \tag{S48}$$

REFERENCES

Bickel, P. J. and Levina, E. (2008). Regularized estimation of large covariance matrices. *The Annals of Statistics* 36, 199–227

Chernozhukov, V., Chetverikov, D., and Kato, K. (2013). Gaussian approximations and multiplier bootstrap for maxima of sums of high-dimensional random vectors. *The Annals of Statistics* 41, 2786–2819

Chernozhukov, V., Chetverikov, D., and Kato, K. (2017). Central limit theorems and bootstrap in high dimensions. *The Annals of Probability* 45, 2309–2352

Leadbetter, M. R., Lindgren, G., and Rootzén, H. (2012). *Extremes and related properties of random sequences and processes* (Springer Science & Business Media)

Li, Z., Qin, S., and Li, Q. (2021). A novel test by combining the maximum and minimum values among a large number of dependent z-scores with application to genome wide association study. *Statistics in Medicine* 40, 2422–2434

Nazarov, F. (2003). *On the Maximal Perimeter of a Convex Set in Rn with Respect to a Gaussian Measure* (Berlin, Heidelberg: Springer Berlin Heidelberg). 169–187. doi:10.1007/978-3-540-36428-3_15

Van der Vaart, A. (1996). Efficient maximum likelihood estimation in semiparametric mixture models. *The Annals of Statistics* 24, 862–878
